# Region-dependent expression and function of integrin α5β1 in protecting against disc degeneration *via* autophagy promotion: an *ex vivo* organ culture model under dynamic mechanical loading

**DOI:** 10.3389/fbioe.2026.1741808

**Published:** 2026-02-24

**Authors:** Mingbin Zhan, Zhen Li, Shuai Chen, Hongkun Chen, Shaozheng Lin, Wentao Sun, Zemin Ling, Peiqiang Su, Shangbin Cui, Xuenong Zou

**Affiliations:** 1 Guangdong Provincial Key Laboratory of Orthopedics and Traumatology/Department of Spinal Surgery, The First Affiliated Hospital of Sun Yat-sen University, Guangzhou, China; 2 AO Research Institute Davos, Davos, Switzerland; 3 Shenzhen Key Laboratory of Bone Tissue Repair and Translational Research, Department of Orthopaedic Surgery, The Seventh Affiliated Hospital of Sun Yat-sen University, Shenzhen, China

**Keywords:** autophagy, degeneration, integrin α5β1, intervertebral disc, mechanical stress

## Abstract

**Introduction:**

Abnormal mechanical loading is a significant pathogenic factor in intervertebral disc degeneration (IVDD), yet the underlying mechanotransduction mechanisms remain incompletely elucidated. This study aimed to investigate the role of integrin α5β1 as a key mechanosensor in regulating the autophagy-apoptosis balance under mechanically induced IVDD.

**Methods:**

Bovine intervertebral discs (IVDs) with intact endplates were cultured in a bioreactor and subjected to dynamic mechanical loading, including physiological loading (PL: 0.02–0.2 MPa, 0.2 Hz) and degenerative loading (DL: 0.32–0.5 MPa, 5 Hz) for 3 and 7 days. Interventions involved the autophagy inhibitor 3-Methyladenine (3-MA), integrin α5β1-specific inhibitory peptide RGD (Arg-Gly-Asp), and the autophagy activator rapamycin. A systematic evaluation was performed, assessing disc height, histomorphology, cell viability, gene/protein expression, autophagy levels, and apoptosis.

**Results:**

Degenerative loading induced progressive IVD degeneration, characterized by irreversible disc height loss, structural disruption, decreased cell viability, and extracellular matrix (ECM) metabolic imbalance. Treatment with 3-MA exacerbated these degenerative changes, confirming the protective role of autophagy. Integrin α5β1 exhibited distinct spatial distribution patterns: its expression was significantly upregulated in the nucleus pulposus (NP) and inner annulus fibrosus (IAF) under degenerative loading, whereas only the β1 subunit was increased in the outer annulus fibrosus (OAF). Functional experiments demonstrated that competitive inhibition of integrin α5β1 by RGD peptide significantly suppressed autophagy activity, exacerbated apoptosis, and promoted ECM degradation. Conversely, rapamycin alleviated degeneration by restoring autophagic flux. Mechanistically, degenerative loading suppressed the FAK/PI3K/AKT/mTOR pathway while upregulating ULK1, and these effects were partially reversed by RGD inhibition.

**Discussion:**

The autophagy-apoptosis balance plays a critical regulatory role in IVDD progression, with integrin α5β1 serving as a crucial upstream mechanosensor that may exert its protective function through modulating the FAK/PI3K/AKT/mTOR pathway. The region-specific distribution of integrin subtypes determines the specificity of mechanotransduction across different disc areas. Targeting the integrin-autophagy axis and its associated signaling pathways may represent a potential therapeutic strategy for mitigating mechanically induced IVDD.

## 1 Introduction

Low back pain (LBP) is one of the most common health problems worldwide, characterized by pain in the low back, sacroiliac region, and buttocks, with or without accompanying radiating pain to the lower extremities (Hall et al., 2019; Urban and Roberts, 2003). Due to its high prevalence, significant disability rates, and substantial medical burden, LBP has garnered considerable attention from researchers worldwide (Hall et al., 2019; Urban and Roberts, 2003). LBP arises from the interaction of multiple complex factors, and IVDD is the primary contributor to its development (Knezevic et al., 2021; Baliga et al., 2015). The IVD, as the body’s load-bearing structure, is primarily composed of three components: the NP in the center surrounded by the anulus fibrosus (AF), and the cartilage endplate (CEP) covering the upper and lower borders. The NP plays a crucial role in maintaining the elasticity of the spine and distributing pressure, while the AF encases and restrains the NP to absorb the pressure and transmit it to the next level. They work synergistically to resist and buffer mechanical stimuli such as gravity and tensile forces (Neidlinger-Wilke et al., 2014).

Aberrant mechanical stress is a primary factor in IVDD (Kos et al., 2019; Ma et al., 2016). As an initial trigger, aberrant mechanical stress can cause localized cumulative damage to the IVD. It leads to a reduction in cell numbers, decreased cellular activity, and dysregulation of ECM metabolism. Ultimately, these changes result in alterations to the microstructure and stress distribution, forming a vicious cycle through a biomechanical-cellular-ECM positive feedback loop, which progressively exacerbates IVDD (Emanuel et al., 2018).

IVDD is characterized by progressive structural failure including matrix degradation and cellular dysfunction, where mechanical overload initiates aberrant mechanotransduction through cytoskeletal remodeling and integrin-mediated signaling (Ke et al., 2021). While integrin α5β1 has been identified as a key mechanosensor responding to abnormal stress through FN-α5β1-RGD interactions (Anderson et al., 2010), its precise role in regulating the autophagy-apoptosis balance remains controversial. Current evidence presents paradoxical findings regarding autophagic activity in IVDD - some studies demonstrate protective functions through damaged organelle clearance (Quan et al., 2020), while others report elevated autophagy markers in advanced degeneration (Wang et al., 2022; Yurube et al., 2021). The spatial distribution and heterodimer formation of integrin α5β1 subunits across IVD regions also remain inadequately characterized (Zhang et al., 2022). This study therefore aims to elucidate the spatiotemporal regulation of integrin α5β1 and its mechanistic link to the autophagy-apoptosis axis during mechanical stress-induced IVDD progression.

Nevertheless, two fundamental aspects remain poorly understood: the dynamic balance between autophagy and apoptosis, and the specific role of integrins as mechanosensors in this process. Elucidating how integrin-mediated mechanical sensing regulates the autophagy-apoptosis axis is crucial for understanding IVDD pathogenesis. This study therefore aims to investigate both the spatiotemporal expression of integrin α5β1 and its role in modulating the autophagy-apoptosis balance during mechanical stress-induced IVDD progression.

## 2 Materials and methods

### 2.1 Isolation and culture of IVDs

Human IVD tissue was obtained from patients diagnosed with lumbar disc herniation, lumbar spondylolisthesis, or lumbar spinal stenosis (n = 12, 7 males, 5 females, age 50.1 ± 13.2 years). Informed consent was obtained from each patient prior to sample collection. This study was approved by the Ethics Committee of the First Affiliated Hospital of Sun Yat-sen University (approval number: [2018]053). The obtained disc samples were promptly embedded in OCT and sectioned into 10 μm frozen slices. Fresh bovine tails were obtained from a local abattoir (Li et al., 2021). Since the bovine disc samples were collected as abattoir leftovers, approval from the ethics committee was not required in accordance with Chinese regulations.

The isolation of IVD whole organs was performed on the first day (Day 0) according to a previously reported method (Zhou et al., 2021). First, soft tissues surrounding the IVDs were carefully removed in a sterile environment. The intact IVDs with their endplate cartilage were rapidly excised with a bandsaw. Blood clots were removed from the endplates using an APEXPULSE Disposable Pulse Lavage System (Apex, Guangzhou, China) with phosphate-buffered saline (PBS). The discs were then sequentially rinsed with PBS containing 10% and 1% penicillin/streptomycin (Gibco, Waltham, MA, USA) for 15 min each. After cleaning, the discs were placed in six-well plates and cultured in Dulbecco’s Modified Eagle’s Medium (DMEM) (Sigma-Aldrich, Munich, Germany) supplemented with 10% fetal bovine serum (FBS, Gibco), 1% penicillin/streptomycin, 1% Insulin-Transferrin-Selenium (ITS, Sigma-Aldrich), 50 μg/mL L-ascorbic acid (Sigma-Aldrich), and 0.1% Primocin (InvivoGen, San Diego, CA, USA). The culture conditions were maintained at 37 °C, 85% humidity, and 5% CO_2_ in an incubator. IVDs from the same bovine tail were randomly assigned to different groups to undergo varying loading time and intensities.

### 2.2 Experiment design and mechanical loading

**FIGURE 1:**
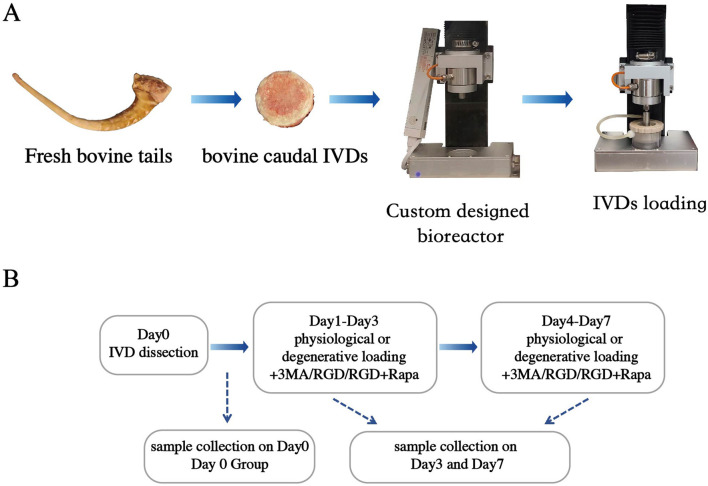
Bioreactor and experimental flow chart. **(A)** The custom-designed bioreactor for physiological and degenerative loading on bovine intervertebral discs (IVDs). **(B)** The experimental design flow chart. RGD: Arg - Gly- Asp; 3-MA: 3-Methyladenine; Rapa: Rapamycin.

IVDs were subjected to dynamic axial loading using a custom-made universal mechanical tester under conditions of 37 °C, 85% humidity, and 5% CO_2_ (Figure 1). The loading was applied as a sinusoidal waveform to simulate physiological or degenerative mechanical loading. Based on previous studies, the following loading parameters were selected: Physiological Loading: 0.02–0.2 MPa, 0.2 Hz, 2 h/day; Degenerative Loading: 0.32–0.5 MPa, 5 Hz, 2 h/day (Secerovic et al., 2022; Lang et al., 2018).

Additionally, 5 mM 3-MA (Selleck Chemicals LLC, Houston, TX, USA) was added to DMEM to investigate the effect of autophagy in the DL + 3-MA group, while the purely physiological and DL groups were cultured in DMEM only. For integrin α5β1 inhibition, the RGD peptide (GRGDSP, 50 μg/mL; MedChemExpress, Monmouth Junction, NJ, USA) was added to the culture medium of the DL + RGD group. In the rescue experiment, both the RGD peptide (50 μg/mL) and rapamycin (5 μM; Selleck Chemicals LLC) were supplemented in the DL + RGD + Rapa group.

After daily loading, the IVDs were transferred to six-well plates containing fresh DMEM and allowed to swell freely in an incubator maintained at 37 °C, 85% humidity, and 5% CO_2_. Samples were collected on Day 0, Day 3 and Day 7 for analysis (Figure 1). Based on the experimental design, the discs were divided into seven groups (Table 1).

**TABLE 1:** IVD dynamic load grouping table.

Group	Loading time (day)		
	0	3	7
PL	Day 0 (no loading)	PL Day 3	PL Day 7
DL		DL Day 3	DL Day 7
DL+ 3-MA		DL + 3-MA Day 3	DL + 3-MA Day 7
DL + RGD		DL + RGD Day 3	DL + RGD Day 7
DL + RGD + Rapa		DL + RGD + Rapa Day 3	DL + RGD + Rapa Day 7

PL: physiological loading, 0.02–0.2 MPa, 0.2 Hz, 2 h/day; DL: degenerative loading, 0.32–0.5 MPa, 5 Hz, 2 h/day.

### 2.3 Measurement of IVD height changes

The height of IVDs was measured at various times during the experiment: Day 0 (baseline): Immediately after sample collection; Post-overnight swelling: After overnight hydration and free swelling in culture medium; Post-mechanical loading: After 2 h of mechanical loading in the bioreactor (Zhou et al., 2021). For each disc, height was measured from two different directions using a vernier caliper, and the values were recorded. The percentage change in disc height was calculated under the following conditions: before and after loading: to assess deformation due to applied mechanical stress; before and after overnight swelling: to evaluate recovery and hydration capacity. These measurements provided insights into the biomechanical responses of IVDs under various loading conditions.

### 2.4 Histology

The IVDs were processed for histological analysis on Days 0, 3, and 7 of the culture periods. The endplates were carefully removed using a scalpel. The NP and AF were separated and rapidly embedded in Optimal Cutting Temperature (OCT) compound (Sakura Finetek U.S.A., Inc., Torrance, CA, USA) and frozen. After that, frozen sections were cut into 10-μm slices, fixed in 100% methanol, and stained with 0.1% Safranin-O (Sigma-Aldrich) and 0.02% Fast Green (Sigma-Aldrich) to visualize the distribution of proteoglycans and collagen fibers. The stained sections were observed and imaged using a digital pathology system (Kfbio, Ningbo, China). A semi-quantitative scoring system (ranging from 0 to 9) was used to assess IVDD scores based on structural integrity and tissue fissures (Shu et al., 2017). The detailed criteria for the grading system are shown in Table 2.

**TABLE 2:** Histological score grading criteria.

Grade	Histological degeneration
IVD structure. Histology cross section clefts characteristics	
0	Normal IVD structure with well-defined annular lamellae, central NP
1	Clefts evidence in inner annulus fibrosus (IAF), normal NP morphology
2	Clefts evident in IAF, mild clefts in outer annulus fibrosus (OAF), inverted IAF lamellae with anomalous distortions
3	Bifurcation/propagation of clefts from IAF into NP margins, mild delamination, or concentric tears between lamellae in IAF.
4	Propagation of cleft into NP, with disruption in normal NP structure, distortion of annular lamellae into atypical arrangements-severe delamination, separation of translamellar cross bridges
Formation of clefts	
0	No clefts in AF
1	Small clefts area in the AF (width of cleft in the range of 90–180 μm)
2	Moderate clefts area in the AF (the number of clefts ≤3, width >180 μm)
3	Moderate clefts area in the AF (the number of clefts >3, width >180 μm)
Clefts direction	
0	Clefts were parallel to the AF lamellae
2	Clefts were perpendicular to the AF lamellae

This histological analysis enabled the assessment of structural changes in IVDs, including proteoglycan depletion and collagen fiber disorganization, across different experimental groups and time points.

### 2.5 Cell viability

Cell viability was evaluated using Lactate Dehydrogenase (LDH) and Ethidium Homodimer (ETH) staining. The staining solution contained Polypep, Glycyl-glycine (Gly-Gly), Lactic acid, Nicotinamide adenine dinucleotide (NAD), Nitroblue tetrazolium (NBT) (all from Sigma-Aldrich), and Ethidium Homodimer-1 (ETH-1, Thermo Fisher Scientific).

Distinct regions within the NP, IAF, and OAF were stained to differentiate between live and dead cells. Cells stained blue or blue/red were considered viable, while cells stained only red were considered non-viable (dead). Random images were captured from each section in the NP, IAF, and OAF regions for analysis. The number of live and dead cells in each field was quantified using ImageJ software (National Institutes of Health, USA). The percentages of live and dead cells were calculated to assess cell viability.

This analysis provided quantitative evidence for cell survival across different regions of the IVDs under different dynamic loading conditions.

### 2.6 RNA extraction and gene expression analysis

RNA was extracted from the NP and AF tissues after culture on Days 0, 3, and 7 for gene expression analysis. Approximately 150 mg of NP and AF tissues were harvested from each sample. The tissues were minced into small fragments and digested in a 2 mg/mL pronase solution for 1 h. After digestion, the tissues were centrifuged and pulverized with liquid nitrogen using a custom-made pestle device. Total RNA was then extracted with Trizol reagent (Invitrogen), and RNA concentration and purity were measured with a spectrophotometer to ensure quality. For consistency, all RNA samples were reverse transcribed into cDNA using the PowerUp SYBR Green Master Mix (Thermo Fisher Scientific, Waltham, MA, USA) on a Real-Time PCR System (Bio-Rad, Hercules, CA, USA). Real-time quantitative PCR (RT-qPCR) reactions were run on a Real-Time PCR System. Primers were designed using Primer 6.0 software (Applied Biosystems, Foster City, CA) and primer sequences are provided in Table 3. The comparative Ct method was performed for relative quantification of target mRNA, and GAPDH was used as the housekeeping gene.

**TABLE 3:** Oligonucleotide primers (bovine) used for quantitative real-time polymerase chain reaction.

Gene	Primer	Sequence (Forward primer/Reverse primer)
ACAN	Primer forward (5′–3′)	5′-CCA ACG AAA CCT ATG ACG TGT ACT-3′
	Primer reverse (5′–3′)	5′-GCA CTC GTT GGC TGC CTC-3′
Col1A1	Primer forward (5′–3′)	5′-AAG GCC AAG AAG AAG ACA TCC C-3′
	Primer reverse (5′–3′)	5′-CGT GGG GAC TTT GGC GTT AG-3′
Col2A1	Primer forward (5′–3′)	5′-GAG CAG CAA GAG CAA GGA CAA GA-3′
	Primer reverse (5′–3′)	5′-GCA GTG GTA GGT GAT GTT CTG AGA G-3′
MMP3	Primer forward (5′–3′)	5′-AAC CTT CCG ATT CTG CTG TTG CTA-3′
	Primer reverse (5′–3′)	5′-GCT TGC GTA TCA CCT CCA GAG T-3′
ADAMTS4	Primer forward (5′–3′)	5′-CCC CAT GTG CAA CGT CAA G-3′
	Primer reverse (5′–3′)	5′-AGT CTC CAC AAA TCT GCT CAG TGA-3′
ADAMSTS5	Primer forward (5′–3′)	5′-TGT GCG GTG ATT GAA GAC GAT GG-3′
	Primer reverse (5′–3′)	5′-TGC TGG TGA GGA TGG AAG ACA TTA AG-3′
Caspase3	Primer forward (5′–3′)	5′-AGA CAG ACA GTG GTG CTG AG-3′
	Primer reverse (5′–3′)	5′-CCA GGA AAA GTA ACC AGG TGC T-3′
Caspase9	Primer forward (5′–3′)	5′-AAT GCC GAT CTG GCC TAT GT-3′
	Primer reverse (5′–3′)	5′-CGC ATC CTC TCA CAG TCG AT-3′
BAX	Primer forward (5′–3′)	5′-ACC​AAG​AAG​CTG​AGC​GAG​TGT​CT-3′
	Primer reverse (5′–3′)	5′-CCC​AGT​TGA​AGT​TGC​CGT​CAG​AAA-3′
CYCS	Primer forward (5′–3′)	5′-ACC AAC ACC GGT ACT TAG GC-3′
	Primer reverse (5′–3′)	5′-ACA TCA CCC ATT TTT AAA TCG TTC T-3′
MAP1LC3A	Primer forward (5′–3′)	5′-TCA GAC CGG CCT TTC AAG C-3′
	Primer reverse (5′–3′)	5′-GCT CGA TTA TCA CCG GGA TTT-3′
Beclin1	Primer forward (5′–3′)	5′-CTT GGG TTA GAG CTA AAG GAG C-3′
	Primer reverse (5′–3′)	5′-ACT GTA TTC CCT TTG ATA CTG AGC-3′
Integrin α5	Primer forward (5′–3′)	5′-TCA TCT ATA TCC TCT ACA AGC TCG G-3′
	Primer reverse (5′–3′)	5′-GCC TTC AAG ACT GGG AGG AAT C-3′
Integrin β1	Primer forward (5′–3′)	5′-ATG GCC GTG AAT GGA CAG A-3′
	Primer reverse (5′–3′)	5′-CTC GGC ACT GAA CAC ATT CTT TAT-3′
FAK	Primer forward (5′–3′)	GAC​AGT​TAC​AAC​GAG​GGC​GTC​AA
	Primer reverse (5′–3′)	GGC​GGG​CAG​AAC​AGG​AAT​G
PIK3CB	Primer forward (5′–3′)	GGC​AGT​GGA​CTC​ACA​GAT​A
	Primer reverse (5′–3′)	GAG​GTT​AAA​CAT​TGG​GTA​AT
AKT1	Primer forward (5′–3′)	CCC​AAC​ACC​TTC​ATC​ATC​CG
	Primer reverse (5′–3′)	GCC​AGC​GAC​ACC​TCC​ATC​T
MAPK1	Primer forward (5′–3′)	CCA​TCG​ACA​TCT​GGT​CCG​TC
	Primer reverse (5′–3′)	GGG​GAT​CCA​AGA​ATA​CCC​AGA
mTOR	Primer forward (5′–3′)	TGG​TGT​GGA​ACT​TGG​GGA​AC
	Primer reverse (5′–3′)	TGA​GAG​AAG​TCC​CGA​CCA​GT
ULK1	Primer forward (5′–3′)	GAG​AAC​ATC​GCC​AAG​TGC​AAG
	Primer reverse (5′–3′)	CAG​TCC​TTT​CGG​CAC​AAC​AG
GAPDH	Primer forward (5′–3′)	5′-TGA TGA CGA GCT TCC CGT TC-3′
	Primer reverse (5′–3′)	5′-TCG GAG TGA ACG GAT TCG G-3′

### 2.7 Apoptosis analysis

Apoptosis was evaluated using TUNEL staining, a method for detecting DNA fragmentation, a hallmark of apoptosis. The TUNEL assay labels the 3′-OH ends of fragmented DNA with fluorescein-tagged dUTP via terminal deoxynucleotidyl transferase (TdT). Sections of IVDs were processed to enhance permeability for staining. TUNEL reaction solution (Roche, Basel, Switzerland) was then applied to the samples. For the negative control group, only 50 μL of fluorescein-labeled dUTP solution was applied, omitting the TdT enzyme. Nuclei were visualized by counterstaining with a mounting medium containing 4,6-diamidino-2-phenylindole (DAPI, Solarbio, Beijing, China). Images were captured from both the NP and AF regions using a fluorescence microscope. The number of TUNEL-positive (apoptotic) cells and total cells was quantified using ImageJ software (Version 1.53k; NIH Bethesda, MD, USA).

This method provided a quantitative assessment of apoptosis levels in different regions of the IVD, helping to evaluate the cellular response under various dynamic loading conditions.

### 2.8 Autophagy analysis

#### 2.8.1 Immunohistochemistry

To analyze autophagy activity in the NP and AF cells, autophagy-related proteins, Microtubule - associated protein light chain 3 (LC3) and Beclin1, two key proteins for autophagosome formation, were examined using IHC (immunohistochemical) and WB. Frozen tissue sections (10 μm) were hydrated and then incubated in 0.6% hydrogen peroxide solution for 15 min to inactivate endogenous peroxidase. 3% BSA (Sigma-Aldrich) was used as the blocking solution, and 0.1% Triton X-100 was used as permeabilization buffer. Sections were incubated with primary antibodies for LC3 (1:400, Abcam, Cambridge, UK, ab128025) and Beclin1 (1:400, Abnova, Taipei, Taiwan, PAB12473) overnight at 4 °C. After washing three times with TBST, sections were incubated with secondary antibodies (Proteintech, Rosemont, IL, USA) at room temperature for 1 h. The sections were then stained with DAB solution (Solarbio) and hematoxylin (Solarbio). Images were captured using a Zeiss LSM 880 with Airyscan (Carl Zeiss AG, Oberkochen, Germany). Random fields of each tissue section were captured and analyzed. Positive areas were quantified with ImageJ software.

#### 2.8.2 Western blotting

Protein extraction and WB were performed to analyze LC3 expression in the NP. On Day 0, 3, and 7, NP samples were harvested, washed three times with cold PBS and pulverized under liquid nitrogen with a custom-made pestle device. Pulverized NP tissues were dissolved in RIPA buffer (Thermo Fisher Scientific) containing a protease inhibitor cocktail (Thermo Fisher Scientific) and PMSF (Boster, Pleasanton, CA, USA). The tissues were ground repeatedly to ensure complete lysis and protein release. After protein extraction and quantification, protein extracts were separated on a 4%–12% Bis-Tris NuPAGE gel (Invitrogen) by electrophoresis. The separated proteins were then transferred onto a PVDF membrane (Invitrogen) and blocked with 5% non-fat milk for 1 h at room temperature. After that, the membranes were incubated overnight at 4 °C with primary antibodies (LC3, Abcam, Cambridge, UK, ab128025; Beclin1, Abnova, Taipei, Taiwan, PAB12473) both at 1:10,000 and then incubated for 1 h at room temperature with HRP-conjugated goat anti-rabbit secondary antibodies (1:10,000, Abcam). Antigen-antibody complexes were visualized using ECL reagents (EpiZyme, Cambridge, MA, USA). Protein bands were detected and quantified using ImageJ software, with GAPDH for standardization of protein expression levels.

### 2.9 Immunofluorescence Co-staining for integrin α5 and β1

To examine the spatial distribution of mechanosensitive integrin subunits, co-localization of integrin α5 and β1 was assessed by immunofluorescence. Frozen sections (10 μm) were permeabilized with 0.5% Triton X-100 and blocked with 1% BSA. Sections were incubated overnight at 4 °C with primary antibodies against integrin α5 (1:400, Usbiological, bs-0486R) and integrin β1 (1:400, Usbiological, C2381-03K), followed by fluorescent secondary antibody incubation. After DAPI counterstaining, images were acquired using a Zeiss LSM 880 microscope. Co-localization analysis was performed on six random fields per section using ImageJ software to quantify co-positive cells and co-localization areas. Negative controls received PBS instead of primary antibodies, and all experiments were performed in triplicate.

### 2.10 Automated Western blot (Wes) analysis

To quantify the expression and phosphorylation levels of core proteins within the FAK/PI3K/AKT signaling pathway in nucleus pulposus (NP) and annulus fibrosus (AF) tissues of the intervertebral disc, Western blot analysis was performed using the automated Wes system. Denatured protein lysates, specific primary antibodies, fluorescently conjugated secondary antibodies, and accompanying reagents were sequentially loaded into the designated wells of the assay plate. The Wes system then automatically performed capillary-based size separation of proteins, target-specific immunolabeling via antibody binding, and chemiluminescent signal detection. The primary antibodies used were as follows: PI3K (ABMART, T40115), AKT (ABMART, T55561), phosphorylated PI3K (p-PI3K; Universal Biologicals, AF3241), and an additional PI3K antibody (ABMART, MC33281). The relative expression level of each target protein was normalized to β-actin, which served as the internal loading control.

### 2.11 Statistical analysis

All statistical analyses were performed using GraphPad Prism 10 software (GraphPad Software, Inc., La Jolla, CA, USA). The normality of data distributions for each group was assessed by the Shapiro-Wilk test. Unpaired Student’s t-test was used for comparisons between two independent groups. Comparisons of multiple groups with a single variable were assessed by one-way ANOVA. When two independent variables were involved, two-way ANOVA with Tukey’s *post hoc* test was used for pairwise comparisons. A p-value < 0.05 was considered statistically significant.

## 3 Result

### 3.1 Autophagy activity in human IVDs

**FIGURE 2:**
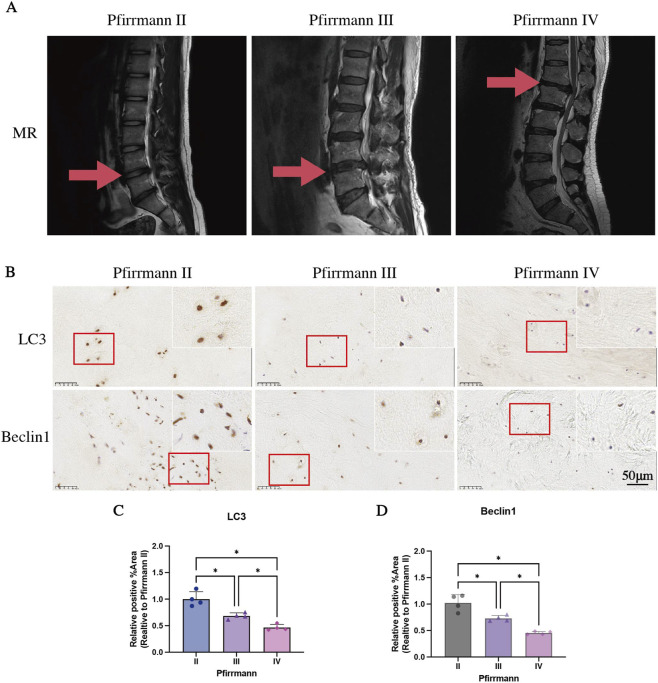
The expression of Microtubule - associated protein light chain 3 (LC3) and Beclin1 in IVDs classified as Pfirrmann grade II to IV. **(A)** Representative T2-weighted MRI images and nucleus pulposus (NP) signal intensity of Pfirrmann grade II, III, and IV according to the Pfirrmann grade classification. **(B–D)** Immunohistochemical staining and statistical analysis of LC3 and Beclin1 in NP tissues across Pfirrmann grade II, III, and IV. Arrows indicate the sample IVD segments. Quantitative analysis (n = 4) reveals significant reductions in LC3 and Beclin1 expression as the degeneration grade increases. Data are expressed as mean ± SD. Scale bar: 50 μm. *p < 0.05.

According to the widely accepted Pfirrmann grading system for IVDD, the collected human IVDs with different degrees of degeneration were classified into Pfirrmann grade II, grade III, and grade IV based on T2-weighted magnetic resonance imaging (MRI) results. The images corresponding to these three grades are shown in Figure 2, representing patients with Pfirrmann grade II, III, and IV (Figure 2A).

IHC analysis revealed a progressive downregulation of autophagy markers in NP tissues with advancing IVD degeneration. LC3 and Beclin1 expression decreased significantly as the severity of degeneration increased (p < 0.05, Figures 2B–D), demonstrating an inverse correlation between autophagic activity and degenerative progression.

### 3.2 Biomechanical, structural, and molecular responses of IVDs to mechanical loading

**FIGURE 3:**
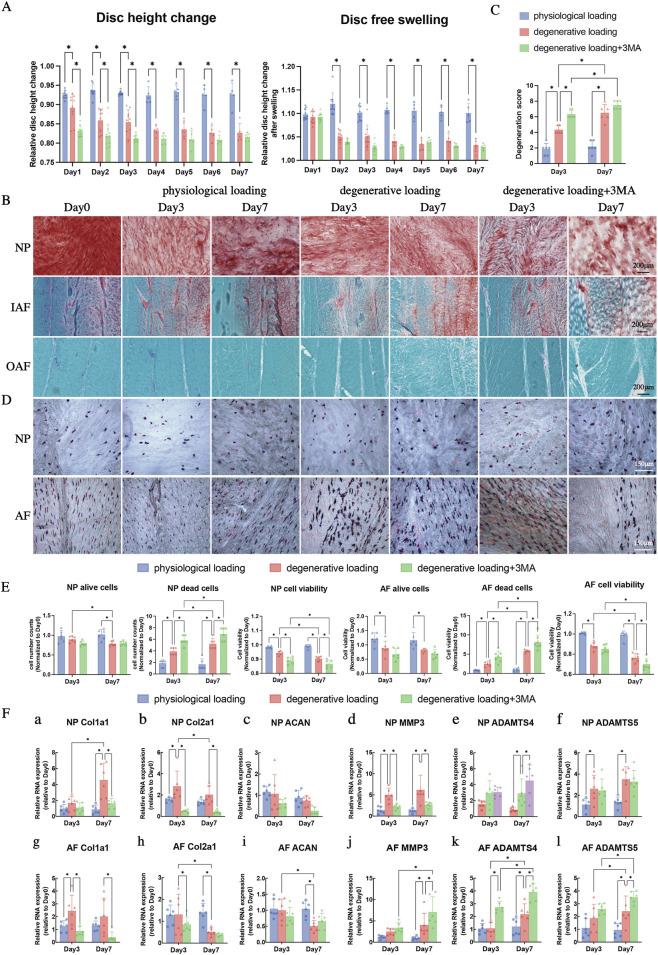
Comprehensive analyses of intervertebral disc (IVD) biomechanical properties, morphology, cell viability, and extracellular matrix (ECM)-related gene expression under physiological and degenerative loading conditions (with/without 3-MA). **(A)** Changes in IVD height and overnight free-swelling recovery from Day 0 to Day 7. **(B)** Representative images of Safranin O/Fast Green staining of the NP, IAF, and OAF on Day 0, Day 3, and Day 7. **(C)** Histological degeneration scores based on Safranin O/Fast Green staining, reflecting structural integrity and fissure formation in IVDs (n = 6). **(D)** Representative images of Lactate Dehydrogenase (LDH)/ethidium homodimer (ETH) staining of the NP, IAF, and OAF on Day 0, Day 3, and Day 7. **(E)** Quantitative analysis of cell viability in NP and anulus fibrosus (AF) tissues: number of alive cells, number of dead cells, and cell viability rate. **(F)** Expression of ECM synthesis-related genes (Col1a1, Col2a1, ACAN) and ECM degradation-related genes (MMP3, ADAMTS4, ADAMTS5) in NP and AF on Day 0, Day 3, and Day 7. All data were normalized to the Day 0 group and expressed as mean ± SD (n = 6). Scale bars: 200 μm (Safranin O/Fast Green staining), 75 μm (LDH/ETH staining). Statistical significance: *p < 0.05. Abbreviations: Col, collagen; ACAN, aggrecan; MMP, matrix metalloproteinases; ADAMTS, a disintegrin and metalloproteinase with thrombospondin motifs.

Comprehensive evaluation across multiple analytical dimensions consistently demonstrated progressive intervertebral disc degeneration under aberrant mechanical stress. Disc height analysis revealed fundamentally distinct biomechanical responses to different loading regimens (Figure 3A). The PL group maintained excellent structural integrity with minimal height loss (6.7% ± 1.7% over 7 days; Figure 3A, p > 0.05 versus baseline) and complete functional recovery following overnight free swelling, indicating preserved fluid exchange mechanisms. In marked contrast, degenerative loading induced significant, progressive height reduction (15.3% ± 3.5%; Figure 3A, p < 0.05) with time-dependent impairment of recovery capacity. The DL + 3-MA group exhibited accelerated early damage progression, showing significantly greater height loss during the initial 3-day period compared to degenerative loading alone (Figure 3A, p < 0.05). Critically, both degenerative groups demonstrated irreversible height loss beyond Day 2, confirming permanent compromise of osmotic rehydration capacity as a fundamental hallmark of advanced matrix degeneration (Figure 3A, p < 0.05 for DL and DL+3-MA versus PL).

Safranin O/Fast Green staining and a modified histological grading system (Shu et al., 2017) were used to evaluate IVD morphology across groups. Histopathological examination provided compelling evidence of progressive structural deterioration (Figures 3B,C). While physiological loading maintained normal tissue architecture throughout the experimental period, degenerative loading induced characteristic pathological progression: initial cleft formation in the nucleus pulposus and irregular fissures in the AF by Day 3, advancing to full-scale AF delamination, concentric tearing, and multidirectional fissuring by Day 7. Autophagy inhibition significantly exacerbated these structural defects, producing more severe and irregular tissue damage that translated to significantly higher degeneration scores at the Day 3 timepoint compared to degenerative loading alone (Figure 3C, p < 0.05).

Cell viability assessment revealed pronounced mechanical load-dependent effects on disc cell survival (Figures 3D,E). Physiological loading maintained excellent cellular viability rate in both disc regions, while degenerative loading triggered substantial cell death with region-specific vulnerability - AF cells demonstrated earlier and more pronounced viability loss compared to NP cells. Autophagy inhibition through 3-MA treatment significantly amplified these detrimental effects, further reducing viability in both disc regions and increasing dead cell counts, particularly in the AF (Figures 3D,E, p < 0.05).

Molecular analysis delineated the regulatory dynamics underlying extracellular matrix homeostasis (Figure 3F). Degenerative loading induced a biphasic transcriptional response: early compensatory upregulation of anabolic genes (Col2a1 in nucleus pulposus: 3.5-fold; Col1a1 in AF: 2.5-fold) on Day 3, followed by a decisive shift to catabolic dominance by Day 7 characterized by significant Col2a1 downregulation and coordinated upregulation of matrix-degrading enzymes (matrix metalloproteinases 3 (MMP3), a disintegrin and metalloproteinase with thrombospondin motifs 4 (ADAMTS4), a disintegrin and metalloproteinase with thrombospondin motifs (ADAMTS5)) in both disc regions. Autophagy inhibition markedly exacerbated these degenerative transcriptional patterns, amplifying matrix gene downregulation (NP and AF: Col1a1, Col2a1, Figure 3F, p < 0.05) while enhancing catabolic pathway activation (NP and AF: MMP3, ADAMTS4, ADAMTS5, Figure 3F, p < 0.05).

These comprehensively integrated findings demonstrate that aberrant dynamic loading induces progressive IVD degeneration through coordinated deterioration across biomechanical, structural, cellular, and molecular domains. The consistent exacerbation of damage through autophagy inhibition provides compelling evidence for its essential protective function in maintaining disc homeostasis under mechanical stress conditions, suggesting its potential as a therapeutic target for intervention in mechanically-induced disc degeneration.

### 3.3 Autophagy and apoptosis responses in IVDs under mechanical loading

**FIGURE 4:**
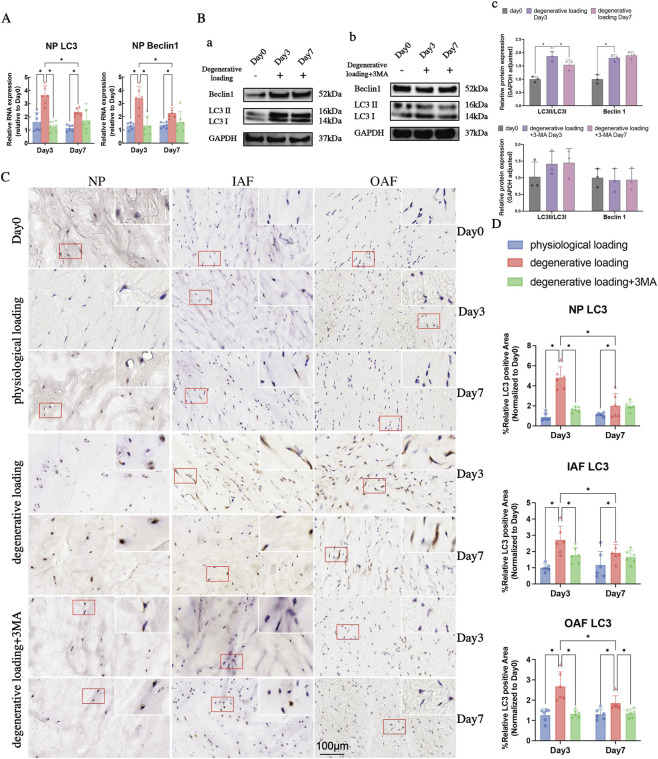
Analyses of autophagy in IVDs under physiological and degenerative loading conditions (with/without 3-MA). **(A)** Quantitative real-time PCR (qPCR) analysis of autophagy-related genes (LC3 and Beclin1) in NP tissues. **(B)** Western blot (WB) analysis of LC3 (LC3-I/LC3-II ratio) and Beclin1 protein expression in the NP of DL and DL+3-MA groups; GAPDH served as a loading control. **(C,D)** Representative IHC staining images **(C)** and quantitative analysis **(D)** of LC3 expression in the NP, IAF, and OAF regions. Red boxes indicate magnified regions. Scale bar: 100 μm. All data were normalized to the Day 0 group and expressed as mean ± SD (n = 6). Statistical significance: *p < 0.05. Abbreviations: LC3, microtubule-associated protein 1 light chain 3.

**FIGURE 5:**
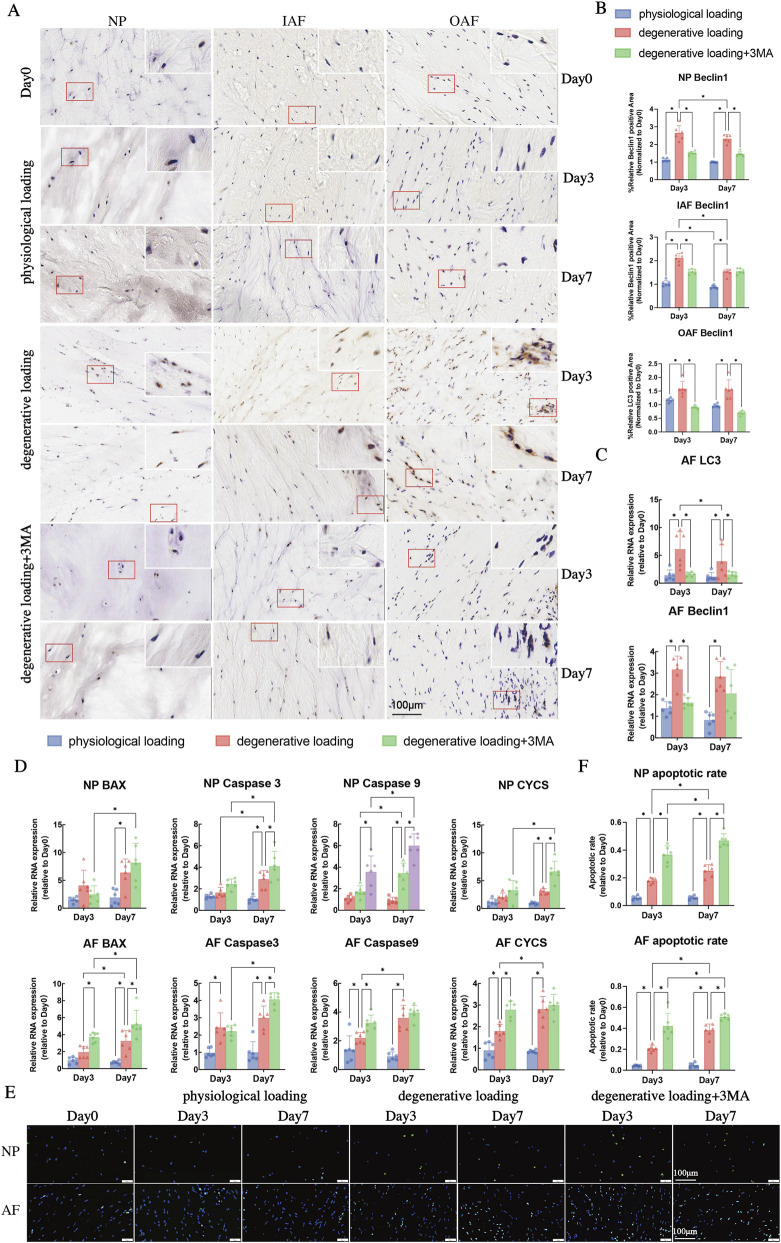
Analyses of autophagy and apoptosis in IVDs under physiological and degenerative loading conditions (with/without 3-MA). **(A,B)** Representative IHC staining images and quantitative analysis of Beclin1 in the NP, IAF, and OAF regions. Red boxes indicate magnified regions. Scale bar: 100 μm. **(C)** Quantitative real-time PCR (qPCR) analysis of autophagy-related genes (LC3 and Beclin1) in AF tissues. **(D)** qPCR analysis of apoptosis-related genes (BAX, Caspase3, Caspase9, CYCS) in NP and AF tissues. **(E)** Representative TUNEL staining images of NP and AF in different groups. Apoptotic cells are stained green. Scale bar: 100 μm. **(F)** Quantitative analysis of apoptosis rates in NP and AF regions from TUNEL staining. All data are normalized to the Day 0 group and expressed as mean ± SD (n = 6). Statistical significance: *p < 0.05. Abbreviations: BAX, Bcl-2 associated X protein; CYCS, cytochrome c.

Analysis of autophagy-related gene expression revealed that degenerative loading significantly upregulated LC3 and Beclin1 in both NP and AF on Day 3 and Day 7 compared to physiological loading (Figures 4A, 5C, p < 0.05). However, when compared within the DL group, their expression levels were significantly lower on Day 7 than on Day 3 (Figures 4A, 5C, p < 0.05), except for Beclin1 in AF (Figure 5C, p > 0.05). Autophagy inhibition with 3-MA significantly suppressed this response, downregulating both LC3 and Beclin1 in NP and AF on Day 3, and LC3 in AF on Day 7 compared to degenerative loading alone (Figures 4A, 5C, p < 0.05).

Protein-level analysis through IHC demonstrated that degenerative loading significantly increased LC3 (Figures 4C,D) and Beclin1 (Figures 5A,B) expression in NP, IAF, and OAF on Day 3 and Day 7 compared to Day 0 (Figures 4D, 5B, p < 0.05). Western blot analysis confirmed significantly increased Beclin1 expression and LC3-II/I ratio in NP under degenerative loading on both Day 3 and Day 7 (Figure 4B, p < 0.05). Autophagy inhibition with 3-MA significantly reduced LC3 and Beclin1 expression in NP and IAF on Day 3 (Figure 4B, p < 0.05), and decreased LC3-II/I ratio in NP on Day 7 (Figure 4B, p < 0.05). In the DL group, the expression levels of LC3 in the nucleus pulposus and inner/outer AF, as well as Beclin1 in the inner AF, all demonstrated a downregulation on day 7 compared to day 3 (Figures 4D, 5B, p < 0.05). IHC analyses revealed that autophagy activity peaked on Day 3 before declining by Day 7 in the DL group (Figures 4D, 5B, p < 0.05).

Analysis of apoptosis-related genes showed that degenerative loading significantly upregulated Caspase3, Caspase9, and cytochrome c (CYCS) in AF on Day 3 compared to physiological loading (Figure 5D, p < 0.05), while no significant changes were observed in NP (Figure 5D, p > 0.05). By Day 7, however, Caspase3, Caspase9, Bax, and CYCS were significantly upregulated in both NP and AF (Figure 5D, p < 0.05). The DL + 3-MA group exhibited enhanced apoptotic activation, with significant upregulation of Caspase9, Bax, and CYCS in AF and Caspase9 in NP on Day 3, and upregulation of Caspase3, Caspase9, CYCS in NP and Caspase3, Bax in AF on Day 7 compared to degenerative loading alone (Figure 5D, p < 0.05).

TUNEL staining analysis (Figure 5E) with quantitative assessment (Figure 5F) showed minimal TUNEL-positive cells in both Day 0 and PL groups, with no significant differences between them (Figures 5E,F, p > 0.05). Degenerative loading significantly increased apoptotic rates in both NP and AF on Day 3 and Day 7 compared to physiological loading (Figures 5E,F, p < 0.05). Autophagy inhibition further exacerbated apoptosis, with significantly higher TUNEL-positive cell rates in both NP and AF compared to degenerative loading alone (Figures 5E,F, p < 0.05). Both degenerative groups showed significantly higher apoptosis on Day 7 than on Day 3 (Figures 5E,F, p < 0.05).

These integrated results demonstrate that degenerative loading induces early autophagy activation that peaks at Day 3 followed by decline, while apoptosis shows progressive escalation. Autophagy inhibition suppresses the autophagic response while amplifying apoptotic cell death, confirming autophagy’s protective role against mechanical stress-induced apoptosis.

### 3.4 Spatiotemporal expression patterns of integrin α5β1 in IVDs under mechanical loading

**FIGURE 6:**
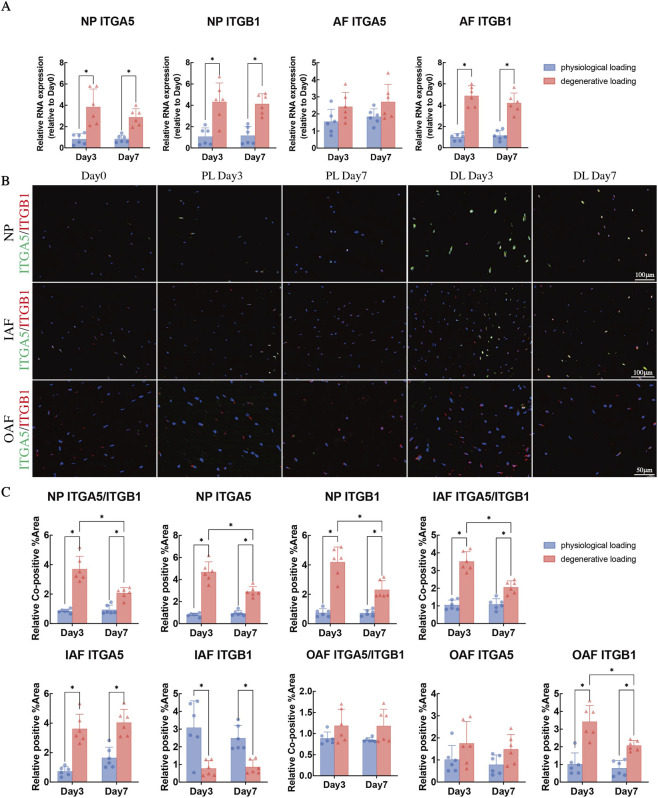
Analysis of integrin α5, integrin β1, and fibronectin in NP and AF tissues under physiological and degenerative loading conditions. **(A)** Quantitative real-time PCR (qPCR) analysis of integrin α5, integrin β1 in NP and AF across experimental groups. **(B)** Representative immunofluorescence double-staining images of integrin α5 (red fluorescence) and integrin β1 (green fluorescence) in NP, IAF, and OAF. Cell nuclei were counterstained with DAPI (blue). **(C)** Quantitative analysis of immunofluorescence staining: co-positive stained area of integrin α5 and β1, positive-stained area of integrin α5, and positive -stained area of integrin β1 in NP, IAF, and OAF, respectively. All data were normalized to the Day 0 group and expressed as mean ± SD (n = 6). Statistical significance: *p < 0.05. Abbreviations: ITGA5, integrin α5; ITGB1, integrin β1.

qPCR results indicated that under degenerative loading, the mRNA levels of integrin α5 and β1 were significantly upregulated in the NP on both day 3 and day 7 (Figure 6A, p < 0.05). In the AF, integrin β1 mRNA expression was upregulated, while no significant change was observed for α5 (Figure 6A, p > 0.05).

Immunofluorescence analysis further delineated the spatiotemporal expression patterns of integrin α5 and β1 subunits in response to mechanical loading. Under physiological loading, both subunits exhibited stable membrane localization across all IVD regions, with expression levels comparable to the Day 0 group (Figures 6B,C). In contrast, degenerative loading induced region-specific alterations. In the NP, the co-expression level of integrin α5 and β1 was significantly increased on day 3, with the co-positive area ratio being 3.7-fold that of the Day 0 group (Figure 6C, p < 0.05). While this elevated expression persisted until day 7, it was significantly downregulated compared to day 3 (Figure 6C, p < 0.05). In the IAF, the co-localization of α5β1 was also significantly enhanced on day 3 but markedly decreased by day 7 (Figure 6C, p < 0.05). Furthermore, the number of β1 single-positive cells was higher in the IAF than in the NP. In the OAF, degenerative loading significantly upregulated the expression of the β1 subunit (Figure 6C, p < 0.05). However, the expression of the α5 subunit and the co-localization level of α5β1 did not show a significant increase (Figure 6C, p > 0.05), suggesting that mechanical signaling in this region may be mediated by other β1-containing integrin heterodimers.

Based on our temporal analysis, a striking parallel emerged: under degenerative loading, the activity of autophagy (as indicated by LC3 and Beclin1) and the expression of integrin α5β1 in the NP and IAF peaked synchronously on day 3, followed by a concurrent decline by day 7. This spatiotemporally coordinated pattern strongly suggests that integrin α5β1 acts not merely as a passive mechanosensor, but rather as an upstream signaling hub that actively triggers a protective autophagic flux in response to mechanical perturbation during the early stage. The subsequent downregulation of both components likely contributes to the insufficiency of the pro-survival mechanism, ultimately shifting cellular fate toward apoptosis. To directly test the hypothesis that integrin α5β1 activation serves as an upstream driver for autophagy induction, we employed a combined loss-of-function and rescue strategy. Specifically, we utilized the RGD peptide to competitively inhibit integrin α5β1, assessing whether this suppression directly impairs autophagy activation and exacerbates disc degeneration. Conversely, we applied rapamycin to bypass integrin signaling and directly stimulate autophagy, examining its potential to counteract the degenerative effects induced by integrin blockade, thereby establishing a causal relationship within this mechanotransduction pathway.

### 3.5 RGD peptide suppresses autophagy activation in IVDs

**FIGURE 7:**
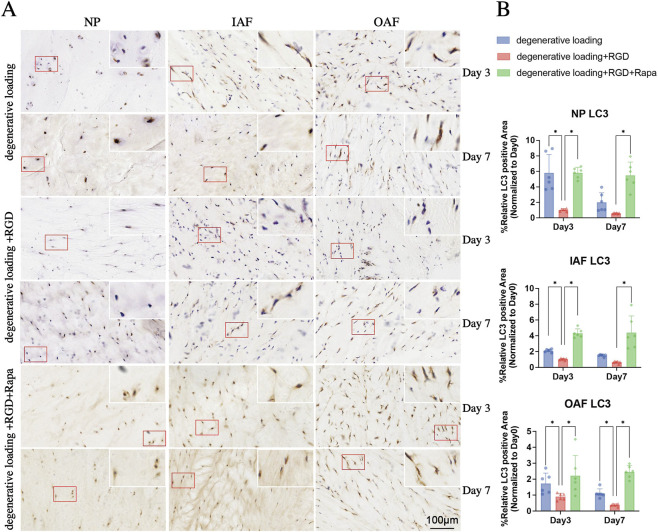
Analysis of autophagy in IVDs under degenerative loading with RGD and rapamycin intervention. **(A,B)** Representative immunohistochemical (IHC) staining images **(A)** and quantitative analysis **(B)** of LC3 in NP, IAF, and OAF regions. Experimental groups include degenerative loading (DL) Day3, DL Day7, DL + RGD Day3, DL + RGD Day7, DL + RGD + Rapa Day3, and DL + RGD + Rapa Day7. Red boxes indicate magnified regions. Scale bar: 100 μm. All data were normalized to the Day 0 group and expressed as mean ± SD (n = 6). Statistical significance: *p < 0.05.

**FIGURE 8:**
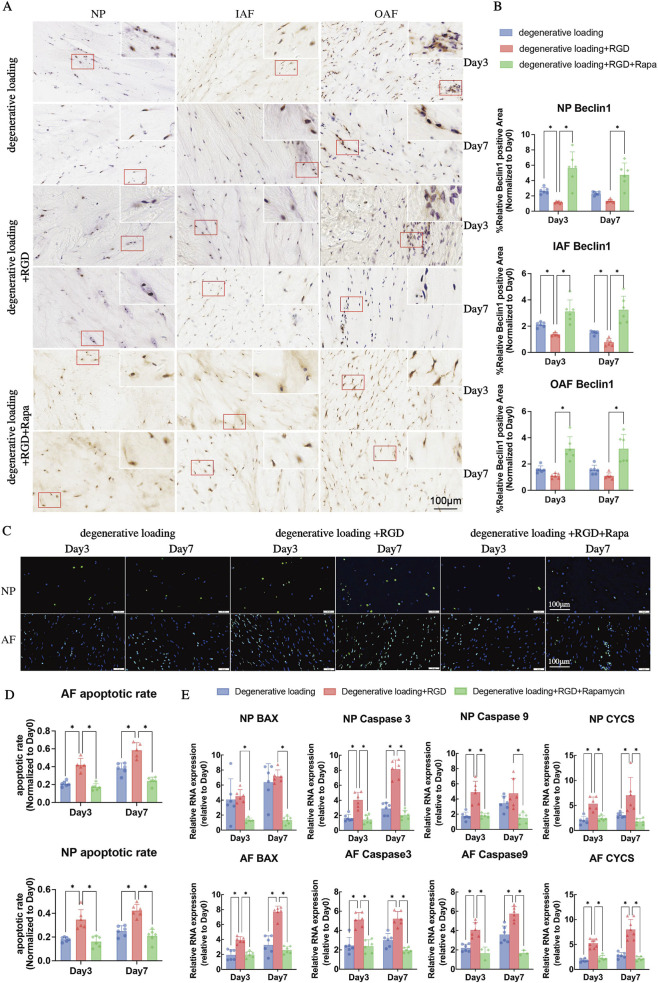
Analysis of autophagy and apoptosis in IVDs under degenerative loading with RGD and rapamycin intervention. **(A,B)** Representative immunohistochemical IHC staining images and quantitative analysis of Beclin1 in NP, IAF, and OAF regions. Scale bar: 100 μm. **(C)** Representative TUNEL staining images of NP and AF cells. TUNEL-positive (apoptotic) cells are visualized by green fluorescence; cell nuclei are counterstained with DAPI (blue). Scale bar: 100 μm. **(D)** Quantitative analysis of apoptosis rates in NP and AF regions from TUNEL staining. **(E)** Quantitative real-time PCR (qPCR) analysis of apoptosis-related genes (BAX, Caspase3, Caspase9, CYCS) in NP and AF tissues. All data were normalized to the Day 0 group and expressed as mean ± SD (n = 6). Statistical significance: *p < 0.05.

To evaluate autophagy activation in response to RGD challenge and rapamycin intervention, we performed immunohistochemical staining for LC3 and Beclin1 in the NP, IAF, and OAF (Figures 7A,B, 8A,B). In the DL group, moderate expression of LC3 and Beclin1 was observed across all regions at days 3 and 7. RGD treatment (DL + RGD group) significantly reduced the protein levels of both LC3 and Beclin1 in the NP, IAF, and OAF at both time points (Figures 7B, 8B, p < 0.05 vs. DL group), indicating a substantial impairment of autophagy activation. In contrast, rapamycin co-treatment (DL + RGD + Rapa group) markedly elevated the expression of these autophagy-related proteins compared to the DL + RGD group (Figures 7B, 8B, p < 0.05), demonstrating its efficacy in restoring autophagy flux.

Cell apoptosis was assessed by TUNEL staining in the NP and AF (Figures 8C,D). The DL group exhibited a low baseline level of apoptosis at days 3 and 7. RGD treatment significantly increased the apoptotic rate in both the NP and AF compared to the DL group at both time points (Figure 8D, p < 0.05). Notably, the addition of rapamycin (DL + RGD + Rapa group) significantly reduced the number of TUNEL-positive cells relative to the DL + RGD group (Figure 8D, p < 0.05), indicating a potent anti-apoptotic effect.

Furthermore, we analyzed the expression of key apoptosis-related genes in NP and AF (Figure 8E). In the NP, the DL + RGD group significantly upregulated the expression of Caspase 3, Caspase 9, and CYCS at day 3, and Caspase 3 and CYCS at day 7 compared to the DL group (Figure 8E, p < 0.05). In the AF, the DL + RGD group significantly enhanced the expression of BAX, Caspase 3, Caspase 9, and CYCS at both day 3 and day 7 (Figure 8E, p < 0.05 vs. DL group). Rapa co-treatment significantly downregulated the expression of BAX, Caspase 3, Caspase 9, and CYCS in both the NP and AF compared to the DL + RGD group (Figure 8E, p < 0.05), confirming that rapamycin attenuates apoptosis by modulating these critical pro-apoptotic genes.

### 3.6 RGD peptide exacerbates while rapamycin mitigates disc degeneration

**FIGURE 9:**
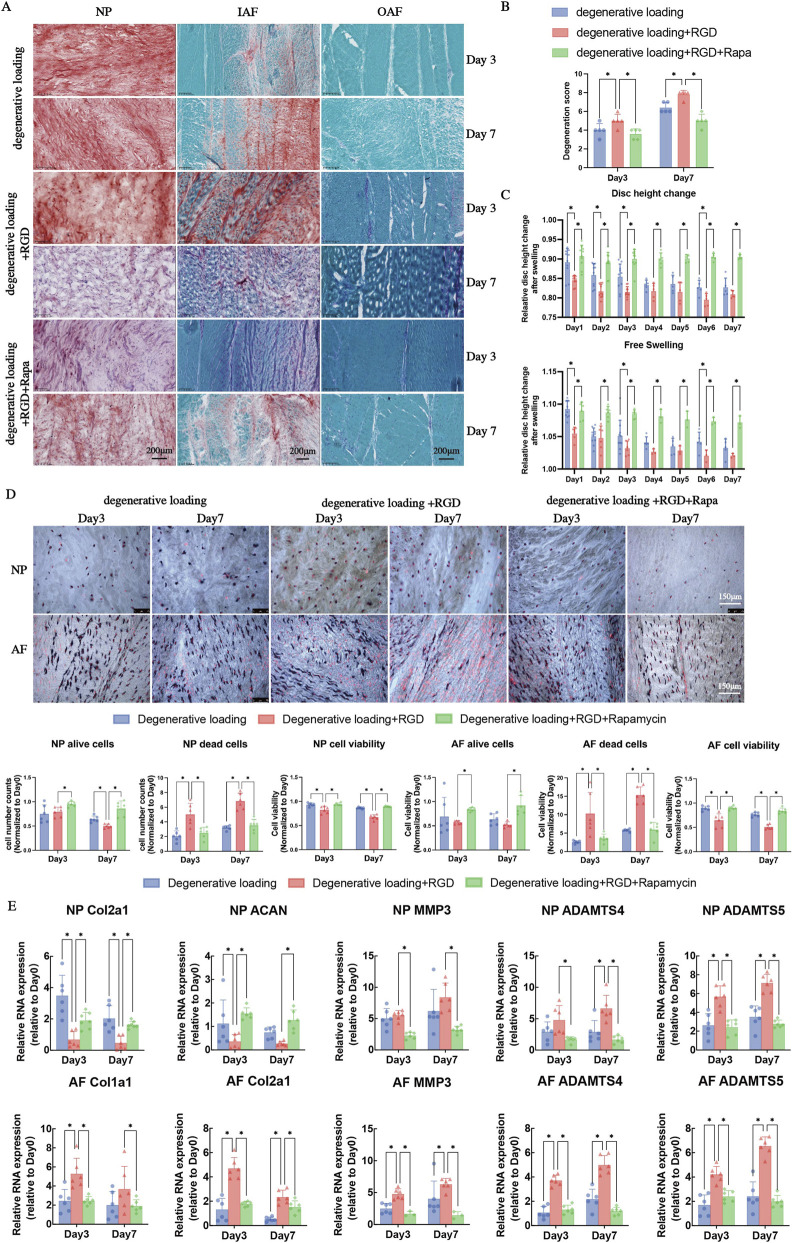
Analyses of intervertebral disc (IVD) structure, cell viability, and ECM metabolism under degenerative loading with RGD and rapamycin intervention. **(A)** Representative Safranin O/Fast Green staining images of the nucleus pulposus (NP), IAF, and OAF in groups: degenerative loading (DL, Day 3, Day 7), DL + RGD (Day 3, Day 7), and DL + RGD + rapamycin (Day 3, Day 7). Scale bar: 200 μm. **(B)** Histological degeneration scores derived from Safranin O/Fast Green staining, reflecting IVD structural integrity. **(C)** Changes in IVD height and overnight free-swelling recovery from Day 1 to Day 7. **(D)** LDH)/ETH staining and quantitative analysis of cell viability in NP and AF: number of alive cells, dead cells, and cell viability rate. In LDH/ETH images, alive cells appear blue/blue-red, and dead cells appear red. Scale bar: 150 μm. **(E)** Quantitative real-time PCR (qPCR) analysis of ECM synthesis-related genes (Col2a1, ACAN) and ECM degradation-related genes (MMP3, ADAMTS4, ADAMTS5) in NP and AF. All data are normalized to the Day 0 group and presented as mean ± SD (n = 6). Statistical significance: *p < 0.05.

Histological assessment of IVD architecture revealed distinct treatment effects (Figure 9A). RGD peptide administration markedly aggravated degenerative changes, producing extensive irregular clefts in the NP and substantially increased fissuring with concentric delamination in the AF. Conversely, rapamycin co-treatment effectively counteracted these RGD-induced alterations, maintaining significantly improved tissue integrity with minimal fissuring compared to the RGD-treated discs.

Histological scoring quantitatively confirmed these observations (Figure 9C). The RGD-treated group demonstrated significantly higher degeneration scores than the DL controls at both day 3 and day 7 (Figure 9C, p < 0.05). Importantly, rapamycin supplementation substantially reduced these scores compared to the RGD-only group (Figure 9C, p < 0.05), demonstrating its potent protective capacity against RGD-accelerated structural deterioration.

Disc height analysis revealed pronounced treatment-specific effects on biomechanical function (Figure 9B). RGD treatment significantly exacerbated disc height loss compared to DL controls, particularly evident on days 1, 2, 3, and 6 (Figure 9B, p < 0.05). Notably, rapamycin co-treatment effectively preserved disc height, showing significantly attenuated reduction throughout the experimental period compared to the RGD-only group (Figure 9B, p < 0.05), indicating its ability to mitigate RGD-induced biomechanical compromise.

Cell viability assessment demonstrated RGD’s detrimental effects and rapamycin’s therapeutic potential (Figure 9D). RGD treatment caused a dramatic increase in cell death throughout NP and AF regions, accompanied by significantly compromised viability (Figure 9D, p < 0.05 vs. DL group). Rapa in intervention successfully reversed this damage, restoring viability parameters to near-normal levels (Figure 9D, p < 0.05 vs. RGD-only group), confirming its efficacy in rescuing RGD-induced cellular demise.

Gene expression analysis revealed distinct region-specific responses to RGD challenge (Figure 9E). In the NP, RGD treatment suppressed anabolic markers (Col2a1, ACAN) while enhancing catabolic factor ADAMTS5 at day 3 (Figure 9E, p < 0.05 vs. DL). By day 7, this metabolic imbalance progressed with sustained Col2a1 suppression and additional ADAMTS4 upregulation (Figure 9E, p < 0.05). In the AF, RGD treatment simultaneously upregulated both anabolic (Col1a1, Col2a1) and catabolic genes (MMP3, ADAMTS4/5) at both timepoints (Figure 9E, p < 0.05), indicating a complex remodeling program potentially contributing to AF disruption.

### 3.7 Integrin α5β1 mediates degenerative-loading-induced modulation of the FAK/PI3K/AKT/mTOR pathway

To investigate the regulatory mechanism of integrin downstream signaling pathways under mechanical stress, we analyzed the expression of key genes in the FAK/PI3K/AKT/mTOR pathway in NP and AF tissues of bovine caudal intervertebral disc organ cultures using qPCR. Compared with the PL group, DL significantly downregulated the mRNA levels of FAK, PIK3CB, AKT1, and mTOR in both NP and AF tissues on Day 3 and Day 7 (Supplementary Figures S1A,B, p < 0.05); meanwhile, the expression of the autophagy-initiating gene ULK1 was significantly upregulated by DL (Supplementary Figures S1A,B, p < 0.05). Intervention with the RGD peptide under degenerative loading (DL + RGD group) partially reversed these changes: the expressions of FAK, PIK3CB, AKT1, and mTOR showed varying degrees of recovery (Supplementary Figures S1A,B, p < 0.05), while the expression of ULK1 was significantly suppressed (Supplementary Figures S1A,B, p < 0.05).

To further verify the expression changes of key proteins in the FAK/PI3K/AKT pathway at the protein level, we quantitatively detected the expression and phosphorylation status of PI3K and AKT in NP and AF tissues using Automated Western blot (Wes) analysis (Supplementary Figures S1C,D). Compared with the PL group, DL significantly reduced the expression of activated phosphorylated forms (p-PI3K and p-AKT) of PI3K and AKT in NP and AF tissues on Day 3. Intervention with the RGD peptide under DL (DL + RGD group) partially rescued the reduction in PI3K/AKT phosphorylation levels, which showed a recovery trend compared with the DL group.

These results indicate that degenerative loading may initiate early autophagy by inhibiting the FAK/PI3K/AKT/mTOR signaling axis and activating ULK1; meanwhile, the RGD peptide can partially reverse the inhibition of the FAK/PI3K/AKT/mTOR signaling axis by competitively inhibiting integrin α5β1, further supporting the key role of integrin α5β1 in regulating the FAK/PI3K/AKT/mTOR signaling network and autophagy initiation under mechanical stress.

## 4 Discussion

IVDD progression is closely associated with mechanical loading conditions. Due to the difficulty in procuring intact human IVDs, bovine caudal discs have been established as a suitable model due to their structural and compositional similarities to human discs and their comparable physiological loading profiles (Zhou et al., 2021; Lang et al., 2018). This study utilized an *ex vivo* bovine disc culture model with cyclic loading protocols: 0.02–0.2 MPa at 0.2 Hz to simulate physiological conditions and 0.32–0.5 MPa at 5 Hz to mimic pathological loading (Secerovic et al., 2022; Lang et al., 2018). The degenerative loading model primarily simulates IVDD caused by abnormal mechanical stress, such as heavy manual labor, carrying heavy loads, or athletic activities. Under physiological loading, discs maintained homeostasis through transient height changes with full recovery, preserved cell viability, and stable expression of both anabolic and catabolic genes, confirming the role of mechanical loading in sustaining disc health through nutrient transport and waste removal (Gawri et al., 2014; Chan et al., 2011).

Excessive mechanical stress beyond disc tolerance induced early degenerative changes. Degenerative loading caused progressive, irreversible disc height loss and early matrix disruption, reducing cell viability rate to 94.1% (NP) and 88.0% (AF) by Day 3. Concurrently, compensatory ECM remodeling was observed with significant upregulation of Col2a1 (NP), Col1 (AF) and MMP3 (NP), suggesting adaptive stiffening against mechanical overload. This transient ECM enhancement represents a protective response to resist injury (Korecki et al., 2008; Liang et al., 2022).

However, concurrent MMP3 and ADAMTS5 upregulation indicates early activation of catabolic pathways prior to structural failure, aligning with the clinical “silent degeneration” phase. While Gernot Lang et al. (2018) reported Col2 downregulation and stable gene expression on Day 4, this discrepancy likely reflects a biphasic ECM adaptation mechanism. During initial loading, IVDs exhibit transient anabolic compensation; however, prolonged stress overwhelms this response, shifting metabolism toward catabolic dominance (Chan et al., 2011; Ding et al., 2021).

Extended 7-day degenerative loading exacerbated degeneration, demonstrating irreversible damage through progressive fissure expansion, persistent disc height loss, and reduced cell viability (NP: 90.2%; AF: 76.3%). ECM disruption was evidenced by Col2 downregulation with concurrent upregulation of Col1a1, MMP3, ADAMTS4/5 in both NP and AF. These findings align with studies confirming IVD sensitivity to abnormal loading (Li J. et al., 2022; Paul et al., 2017), where static compression similarly caused irreversible height loss and ECM imbalance in murine models (Li J. et al., 2022), while dynamic loading induced pan-disc damage in caprine models (Paul et al., 2017).

Under mechanical stress, IVDs exhibit a spatiotemporally regulated biphasic autophagy response coordinated with ECM remodeling and apoptotic activation (Yurube et al., 2021; He et al., 2009). Early-phase autophagic enhancement, particularly in the NP, coincided with compensatory ECM anabolism, suggesting adaptive protection. However, prolonged loading led to autophagic decline, catabolic dominance, and significantly elevated apoptosis. These temporal dynamics support a “threshold-buffered degeneration” model wherein transient autophagy activation delays structural deterioration, but its eventual failure triggers irreversible damage.

Under hypoxic and nutrient-deficient conditions, IVD cells maintain basal autophagy to preserve cellular homeostasis (Kritschil et al., 2022). Our study demonstrates a biphasic autophagic response (peak at Day 3, decline by Day 7) under mechanical stress, consistent with clinical observations by Quan et al. (Quan et al., 2020) and experimental models by Li et al. (2018), Zhao et al. (2019), and Khaleque et al. (2024). This early autophagic activation exhibits protective effects, while its subsequent decline aligns with findings from Yurube et al. (2019) showing reduced autophagic activity in advanced degeneration. Although Ma et al. (2013) reported autophagy-mediated cell death under extreme compression, their cellular model lacks the 3D biomechanical environment essential for evaluating NP-AF crosstalk, a limitation addressed in our study. Mechanical overload induces IVDD through ROS generation, catabolic enzyme activation, and inflammation (Li Y. et al., 2022; Kang et al., 2022). Early autophagy counteracts these effects by clearing damaged component, but its decline leads to ECM dysregulation and accelerated apoptosis (Madhu et al., 2025). Our data demonstrate that autophagy inhibition significantly exacerbates mechanical stress-induced apoptosis, confirming the crucial protective role of autophagy in early-stage IVDD.

Mechanical overload promotes IVDD through ROS-mediated damage and catabolic activation (Feng et al., 2017). Early autophagy mitigates these effects by clearing damaged components (Wang et al., 2023), but its subsequent decline leads to ECM dysregulation and accelerated apoptosis (Zhang et al., 2021). Our study demonstrates a biphasic autophagy-apoptosis interplay, where decreasing autophagic activity coincided with elevated apoptosis, and 3-MA inhibition significantly exacerbated mechanical stress-induced cell death. These findings align with previous reports of time-dependent apoptosis under mechanical loading (Zhang et al., 2011; Liu et al., 2023) and the documented sequence of autophagic protection (Khaleque et al., 2024). The anti-apoptotic role of autophagy observed in our study is further supported by existing literature (Liu et al., 2023), confirming that autophagy serves a protective function until exceeding stress thresholds triggers apoptotic ECM collapse.

While our findings establish autophagy’s protective role in early IVDD, some studies report context-dependent detrimental effects. Wu et al. (2014) observed that lactic acidosis concurrently upregulated autophagy and apoptosis while suppressing matrix synthesis, suggesting stress-specific dysregulation. Similarly, Fu et al. (2020) demonstrated that inflammatory pathways can co-opt autophagy to drive ECM catabolism. These contrasting results highlight autophagy’s dualistic nature in IVDD: transient mechanical stress-induced autophagy protects against apoptosis and maintains ECM homeostasis, whereas sustained autophagy under inflammatory/metabolic stress promotes catabolism. However, both contrasting studies employed cellular models lacking the 3D biomechanical environment and AF-NP crosstalk, limiting their generalizability.

Our study reveals distinct region-specific responses in IVDs under degenerative loading. The NP exhibited markedly enhanced autophagic activity compared to the AF, consistent with reported higher baseline autophagy in NP tissues (Yurube et al., 2021). Conversely, the AF demonstrated more pronounced apoptosis, correlating with its relatively limited autophagic capacity. This regional divergence reflects mechanical stress redistribution during dynamic loading, where pressure shifts from NP to AF regions, overloading the AF’s repair threshold (Paul et al., 2017). Furthermore, NP cells show greater sensitivity to loading frequency while AF cells respond more to load magnitude (Maclean et al., 2004), explaining their differential vulnerability. Unlike mild degeneration observed under lower-frequency loading (Wuertz et al., 2009), our high-frequency regimen (5 Hz) induced progressive autophagic decline and apoptotic elevation, suggesting frequency-dependent disruption of cellular homeostasis. This regional divergence in autophagic and apoptotic responses may be attributed to the distinct mechanical microenvironments and cellular phenotypes between the NP and AF.

The intervertebral disc (IVD), as the largest avascular tissue in the human body, relies on mechanotransduction mechanisms to maintain tissue homeostasis within its unique mechanical microenvironment. The mechanotransduction process comprises three key steps: mechanical sensing by receptors, signal conversion, and downstream pathway activation (Ngai et al., 2018). Among these, integrins serve as crucial transmembrane receptors that connect the extracellular matrix to the intracellular cytoskeleton, playing a central role in mechanical signal transduction.

Integrins consist of α and β subunits that regulate their affinity through conformational changes, transitioning from a resting state to a high-affinity state to initiate downstream signaling (Campbell and Humphries, 2011). In IVD tissues, α1, αV, α5 and β1, β3, β5 subunits are highly expressed (Chen et al., 2022). In IVD tissues, α1, αV, α5 and β1, β3, β5 subunits are highly expressed (Chen et al., 2022). Mechanical stress induces real-time cytoskeletal remodeling and facilitates mechano-chemical signal conversion through mediators such as Rho GTPases (Li et al., 2011).

The RGD peptide acts as a specific competitive inhibitor of integrin receptors, effectively antagonizing integrin functions in cell adhesion, growth, and differentiation (Ruoslahti and Obrink, 1996). Previous studies have demonstrated regional heterogeneity of integrin α5β1 expression in healthy IVDs, with high expression levels in the NP and IAF, but relatively lower expression in the OAF (51). This phenomenon is also observed in AF studies: healthy AF cells under mechanical stretching show downregulated ADAMTS4 expression and enhanced FAK phosphorylation, effects that can be inhibited by RGD peptides, whereas degenerated cells lose this responsiveness (Gilbert et al., 2013). Zhang et al. (2016) further demonstrated that cyclic stretch-induced AF cell apoptosis might be associated with decreased integrin β1 expression. However, these studies based on two-dimensional cell culture systems may have limitations due to the lack of a complete cell-matrix mechanical microenvironment.

Our study systematically analyzed integrin expression patterns across different IVD regions in response to abnormal mechanical stimulation. In an organ culture model, short-term degenerative loading significantly enhanced integrin α5β1 expression in the NP and IAF while OAF expression remained significantly lower. As degeneration progressed, integrin expression demonstrated a downward trend. Particularly noteworthy is the unique expression pattern in the OAF region: under degenerative loading conditions, the NP region showed coordinated upregulation of both integrin α5 and β1 subunits, while the OAF region maintained stable α5 subunit expression but significantly upregulated its partner β1 subunit. This region-specific expression difference strongly suggests that different IVD regions employ distinct mechanical sensing mechanisms, with the NP primarily relying on integrin α5β1 heterodimers for mechanotransduction, while the OAF may involve other β1-containing integrins in mechanical signal perception and transduction.

Functional experiments further confirmed the crucial role of integrin α5β1 in mechanotransduction. When RGD peptides were used to competitively inhibit integrin α5β1 function, we observed significant suppression of autophagy activity, accompanied by markedly increased apoptosis and exacerbated IVD degeneration, specifically manifested as increased disc height loss, disrupted extracellular matrix metabolic balance, decreased cell viability, and elevated histological degeneration scores. Conversely, rapamycin-induced autophagy activation partially reversed these degenerative changes. These findings reveal a sophisticated regulatory network among integrins, autophagy, and apoptosis. During early degeneration, integrin-mediated autophagy activation serves as an important compensatory mechanism, maintaining intracellular homeostasis by clearing damaged organelles and abnormal proteins, thereby delaying the apoptosis process. However, as mechanical loading persists, this protective mechanism gradually becomes dysregulated, ultimately leading to cell apoptosis.

In this study, preliminary qPCR analysis of key genes in the FAK/PI3K/AKT/mTOR signaling pathway revealed that abnormal mechanical loading significantly suppressed this pathway, as indicated by the downregulation of FAK, PIK3CB, AKT1, and mTOR mRNA, while concurrently upregulating the autophagy-initiating gene ULK1. These findings were further corroborated at the protein level using automated Western blot (Wes), which showed that degenerative loading markedly reduced the activated, phosphorylated forms of PI3K and AKT (p-PI3K and p-AKT). Together, these results align with the role of integrin α5β1 as an upstream mechanosensor and suggest that mechanical stress may inhibit the FAK/PI3K/AKT/mTOR axis, thereby relieving its negative regulation on autophagy and triggering an early protective autophagic response. Notably, competitive inhibition of integrin α5β1 with the RGD peptide partially reversed the aforementioned alterations in both gene and protein expression, further supporting the regulatory role of integrins in this pathway. These observations are consistent with previous reports: Gilbert et al. (2013) demonstrated that integrin-mediated FAK phosphorylation is essential for the mechanical response in normal AF cells, while Ni et al. (2014) showed that under serum-deprivation conditions, TGF-β1 activates the PI3K/AKT/mTOR pathway, reduces autophagy, and enhances cell survival in rat AF cells. It is noteworthy that although Gilbert et al. (2013) proposed that mechanotransduction in degenerated cells may shift to RGD-insensitive alternative pathways, the present study observed that integrin α5β1 continues to regulate autophagy via this axis in an early-stage degeneration model, which may reflect the dynamic evolution of mechanosensing mechanisms across different degenerative stages. Certainly, the current evidence remains correlative at the tissue level; further investigation is needed to clarify the temporal and causal relationships within the integrin-autophagy axis. Nevertheless, these multi-level data provide new molecular insights into the protective role of the “integrin-autophagy axis” in mechanically induced intervertebral disc degeneration and strengthen the rationale for targeting this pathway as a potential therapeutic strategy.

This study provides the first organ-level evidence supporting the central role of the integrin-autophagy axis in the mechanical protection of the intervertebral disc (IVD), offering a novel perspective for understanding mechanobiology in IVD degeneration. Unlike previous investigations based on two-dimensional cell culture systems (Le Maitre et al., 2009; Gilbert et al., 2013), our organ culture model more closely recapitulates the three-dimensional mechanical microenvironment of IVD cells, thereby yielding more physiologically relevant insights into mechanotransduction. Specifically, our preliminary findings on the FAK/PI3K/AKT/mTOR pathway demonstrate that abnormal mechanical loading suppresses this signaling axis at both the gene and protein levels, which is concomitant with the activation of autophagy. This result mechanistically links integrin-mediated sensing to downstream autophagic activity. Future work should build upon these observations to further elucidate the specific molecular mechanisms by which different integrin subtypes regulate autophagy and to delineate the dynamic evolution of this pathway throughout the progression of IVD degeneration.

This study has several limitations that warrant consideration. First, as a preliminary pilot investigation, the small sample size may restrict statistical power and generalizability of the observed autophagy-apoptosis dynamics. Second, the uniaxial static compression model, while controlled, oversimplifies spinal biomechanics by excluding synergistic contributions from muscles, ligaments, and facet joints. This limitation may overemphasize axial deformations while underestimating shear/torsional stresses critical to AF lamellar damage *in vivo*. To address this, future work should employ multiaxial bioreactors capable of replicating flexion-rotation kinematics and interfacing with finite element analysis for stress mapping (Secerovic et al., 2022). Third, while the *ex vivo* organ culture model preserves essential tissue complexity, it presents inherent challenges for dissecting specific intracellular signaling cascades. While we observed RGD-sensitive gene and protein expression changes in the FAK/PI3K/Akt/mTOR pathway under degenerative loading, direct measurement of autophagic flux—using tools such as the mRFP-GFP-LC3 tandem probe—remains necessary to functionally validate the proposed integrin-autophagy axis. And moreover, the 7-day observation window captures only acute degenerative responses, neglecting long-term adaptive or maladaptive changes. Incorporating extended timepoints (e.g., Days 14, 28) and higher-frequency sampling (e.g., Days 2, 4, 6) would better resolve temporal transitions in autophagy flux and apoptotic activation during the progression of IVDD caused by abnormal mechanical stress. Addressing these aspects in future work will provide a more complete mechanistic understanding and translational relevance.

## 5 Conclusion

Our study demonstrates that the autophagy-apoptosis balance plays a critical regulatory role in intervertebral disc degeneration. Integrin α5β1 serves as a crucial upstream mechanosensor, exerting its protective function by promoting autophagy via modulation of the FAK/PI3K/AKT/mTOR pathway during early mechanically induced degeneration. The region-specific distribution of integrin subtypes determines the specificity of mechanotransduction across different disc areas. Therefore, targeting the integrin-autophagy axis and its associated signaling pathways may represent a potential therapeutic strategy for mitigating mechanically induced disc degeneration.

## Data Availability

The original contributions presented in the study are included in the article/Supplementary Material, further inquiries can be directed to the corresponding authors.
