# Correction: Region-dependent expression and function of integrin α5β1 in protecting against disc degeneration via autophagy promotion: an *ex vivo* organ culture model under dynamic mechanical loading

**DOI:** 10.3389/fbioe.2026.1840370

**Published:** 2026-05-19

**Authors:** Mingbin Zhan, Zhen Li, Shuai Chen, Hongkun Chen, Shaozheng Lin, Wentao Sun, Zemin Ling, Peiqiang Su, Shangbin Cui, Xuenong Zou

**Affiliations:** 1 Guangdong Provincial Key Laboratory of Orthopedics and Traumatology/Department of Spinal Surgery, The First Affiliated Hospital of Sun Yat-sen University, Guangzhou, China; 2 AO Research Institute Davos, Davos, Switzerland; 3 Shenzhen Key Laboratory of Bone Tissue Repair and Translational Research, Department of Orthopaedic Surgery, The Seventh Affiliated Hospital of Sun Yat-sen University, Shenzhen, China

**Keywords:** autophagy, degeneration, integrin α5β1, intervertebral disc, mechanical stress

There was a mistake in Figures 4, 6–8 as published. Due to an inadvertent error during image assembly, incorrect representative images were displayed in Figure 4C for “NP physiological loading Day 7” and “NP degenerative loading + 3 MA Day 7”, in Figure 6B for “OAF degenerative loading Day 7”, in Figure 7A for “OAF degenerative loading + RGD Day 7” and in Figure 8A for “IAF degenerative loading + RGD Day 7”. The corrected Figures 4, 6–8 appear below.

**FIGURE 4:**
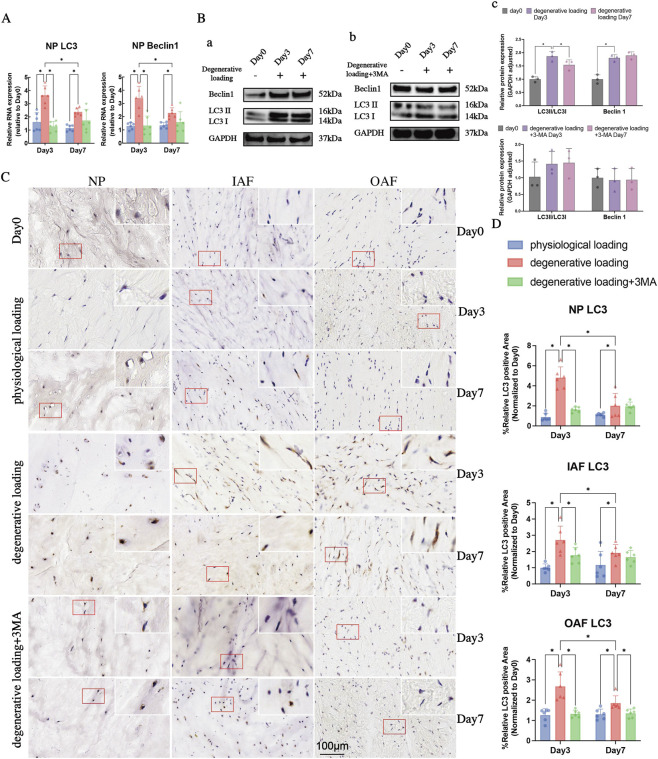
Analyses of autophagy in IVDs under physiological and degenerative loading conditions (with/without 3-MA). **(A)** Quantitative real-time PCR (qPCR) analysis of autophagy-related genes (LC3 and Beclin1) in NP tissues. **(B)** Western blot (WB) analysis of LC3 (LC3-I/LC3-II ratio) and Beclin1 protein expression in the NP of DL and DL+3-MA groups; GAPDH served as a loading control. **(C,D)** Representative IHC staining images **(C)** and quantitative analysis **(D)** of LC3 expression in the NP, IAF, and OAF regions. Red boxes indicate magnified regions. Scale bar: 100 μm. All data were normalized to the Day 0 group and expressed as mean ± SD (n = 6). Statistical significance: *p < 0.05. Abbreviations: LC3, microtubule-associated protein one light chain 3.

**FIGURE 6:**
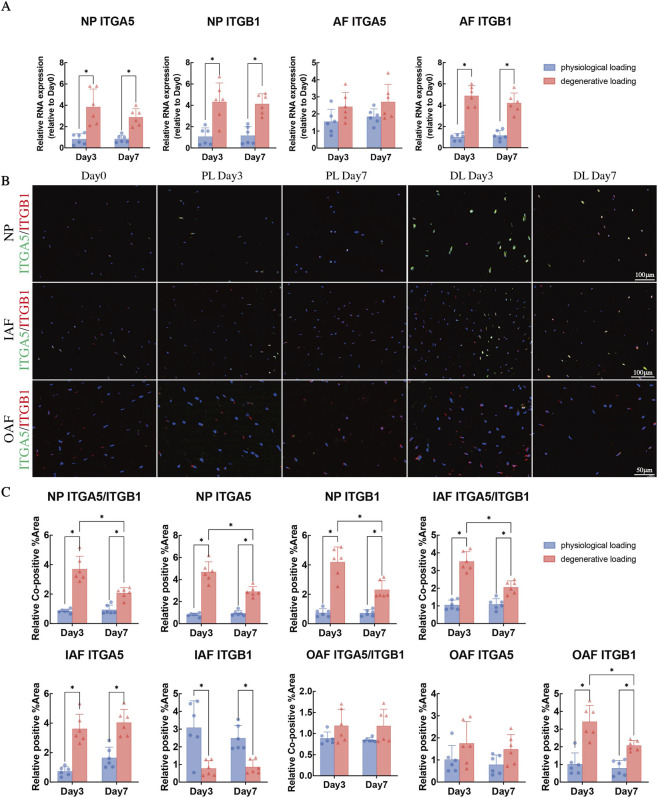
Analysis of integrin α5, integrin β1, and fibronectin in NP and AF tissues under physiological and degenerative loading conditions. **(A)** Quantitative real-time PCR (qPCR) analysis of integrin α5, integrin β1 in NP and AF across experimental groups. **(B)** Representative immunofluorescence double-staining images of integrin α5 (red fluorescence) and integrin β1 (green fluorescence) in NP, IAF, and OAF. Cell nuclei were counterstained with DAPI (blue). **(C)** Quantitative analysis of immunofluorescence staining: co-positive stained area of integrin α5 and β1, positive-stained area of integrin α5, and positive -stained area of integrin β1 in NP, IAF, and OAF, respectively. All data were normalized to the Day 0 group and expressed as mean ± SD (n = 6). Statistical significance: *p < 0.05. Abbreviations: ITGA5, integrin α5; ITGB1, integrin β1.

**FIGURE 7:**
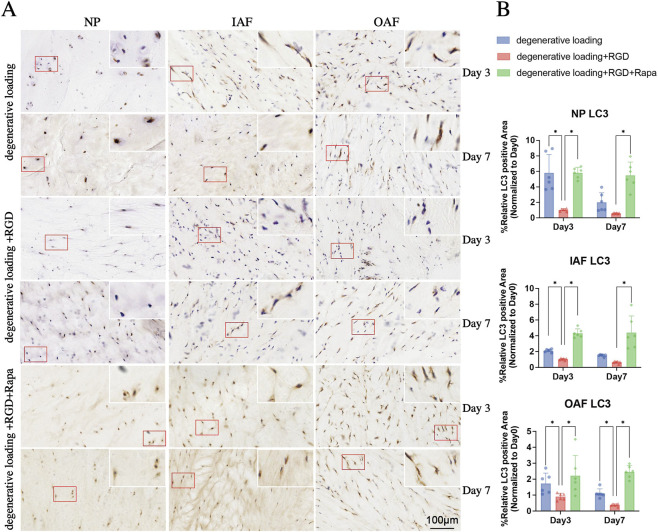
Analysis of autophagy in IVDs under degenerative loading with RGD and rapamycin intervention. **(A,B)** Representative immunohistochemical (IHC) staining images **(A)** and quantitative analysis **(B)** of LC3 in NP, IAF, and OAF regions. Experimental groups include degenerative loading (DL) Day3, DL Day7, DL + RGD Day3, DL + RGD Day7, DL + RGD + Rapa Day3, and DL + RGD + Rapa Day7. Red boxes indicate magnified regions. Scale bar: 100 µm. All data were normalized to the Day 0 group and expressed as mean ± SD (n = 6). Statistical significance: *p < 0.05.

**FIGURE 8:**
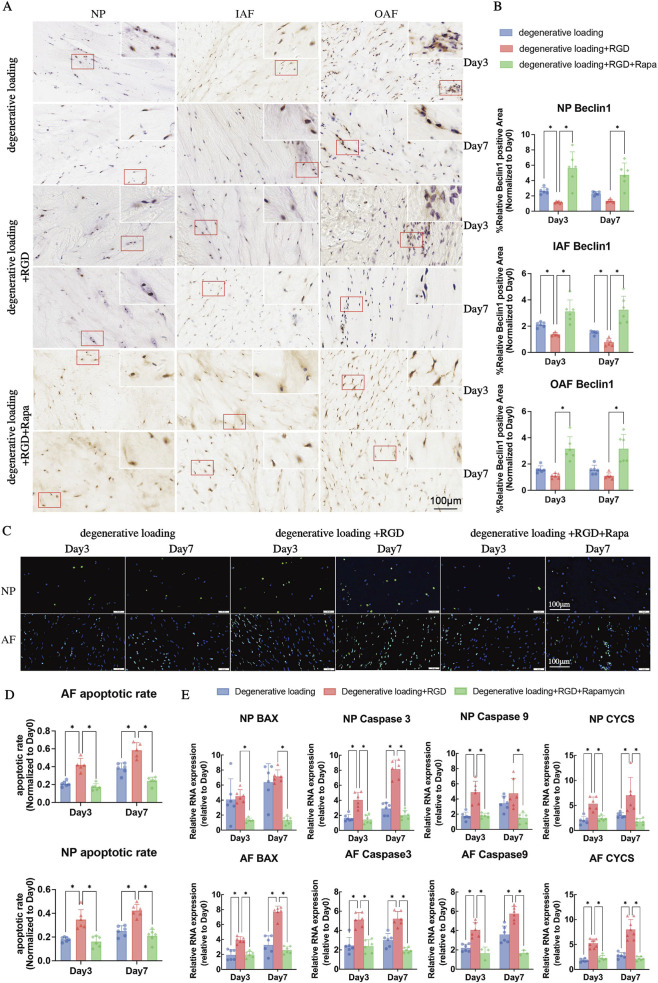
Analysis of autophagy and apoptosis in IVDs under degenerative loading with RGD and rapamycin intervention. **(A,B)** Representative immunohistochemical IHC staining images and quantitative analysis of Beclin1 in NP, IAF, and OAF regions. Scale bar: 100 μm. **(C)** Representative TUNEL staining images of NP and AF cells. TUNEL-positive (apoptotic) cells are visualized by green fluorescence; cell nuclei are counterstained with DAPI (blue). Scale bar: 100 μm. **(D)** Quantitative analysis of apoptosis rates in NP and AF regions from TUNEL staining. **(E)** Quantitative real-time PCR (qPCR) analysis of apoptosis-related genes (BAX, Caspase3, Caspase9, CYCS) in NP and AF tissues. All data were normalized to the Day 0 group and expressed as mean ± SD (n = 6). Statistical significance: *p < 0.05.

The original article has been updated.

