# Supplementary material for: Region-dependent expression and function of integrin α5β1 in protecting against disc degeneration via autophagy promotion: an ex vivo organ culture model under dynamic mechanical loading
Source: Front Bioeng Biotechnol. 2026 Feb 24;14:1741808. doi: 10.3389/fbioe.2026.1741808 (PMC12971973; doi:10.3389/fbioe.2026.1741808)
Supplement: Supplementary file 1 [file Supplementaryfile1.docx]

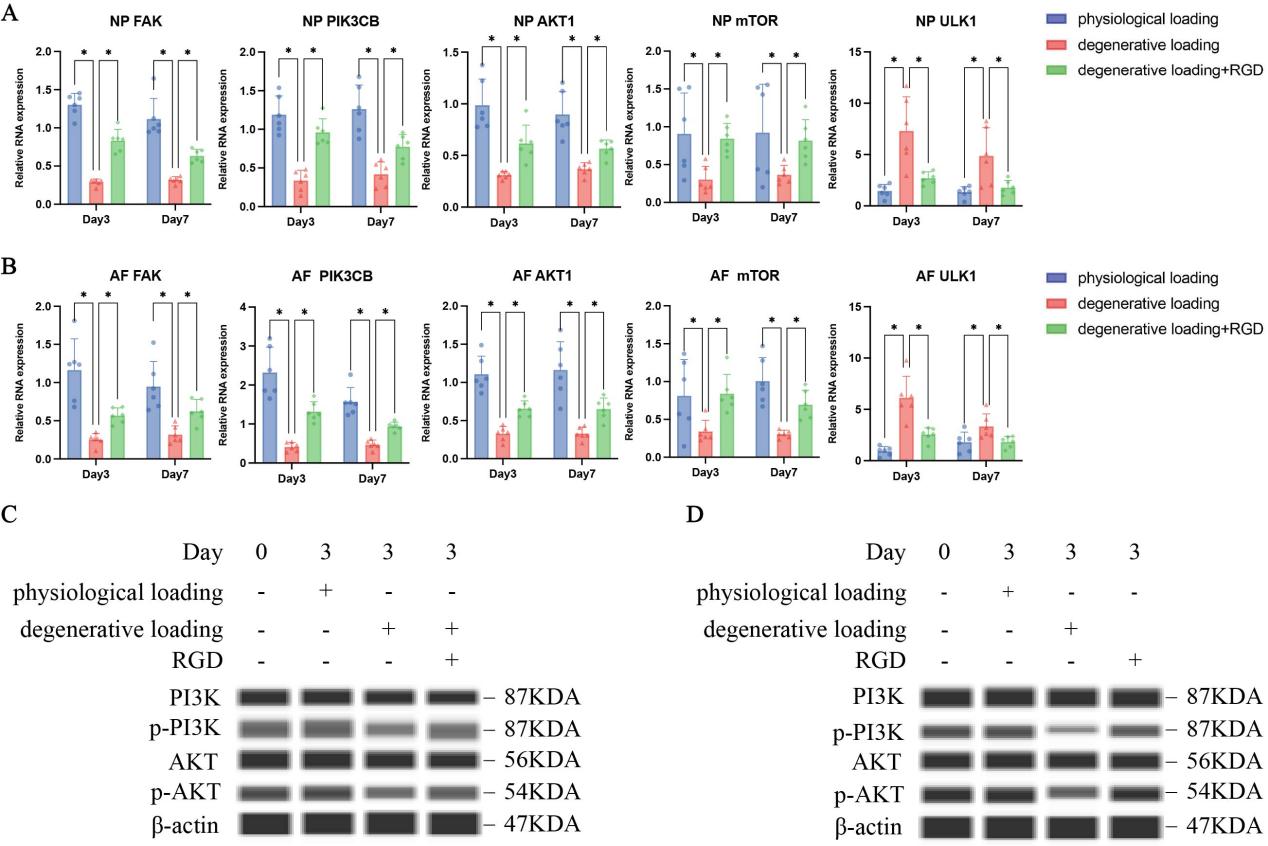


**Supplementary Figure 1. Gene and protein expression analyses in NP and AF tissues of IVDs under Day0, physiological/degenerative loading conditions (with or without RGD).** (A) qPCR analysis of the relative mRNA expression of FAK, PIK3CB, AKT1, mTOR, and ULK1 in IVD NP tissues at Day 3 and Day 7, across three experimental groups: Day0, PL, DL, and DL+RGD. (B) Corresponding qPCR analysis of the above genes in AF tissues at Day 3 and Day 7. (C, D) Automated Western blot (Wes) analysis of protein expression (and phosphorylation) of key PI3K pathway components (PI3K, phosphorylated PI3K (p-PI3K), AKT, phosphorylated AKT (p-AKT)) in IVD NP (C) and AF (D) tissues under different treatment conditions (Day 0, Day 3 with PL/DL/DL+RGD). β-actin was used as the internal reference. All qPCR data are presented as mean ± SD (n = 6). Statistical significance (vs. the corresponding control group): *p < 0.05. Abbreviations: FAK (focal adhesion kinase); PIK3CB (phosphatidylinositol-4,5-bisphosphate 3-kinase catalytic subunit beta); AKT1 (AKT serine/threonine kinase 1); mTOR (mechanistic target of rapamycin kinase); ULK1 (unc-51 like autophagy activating kinase 1).


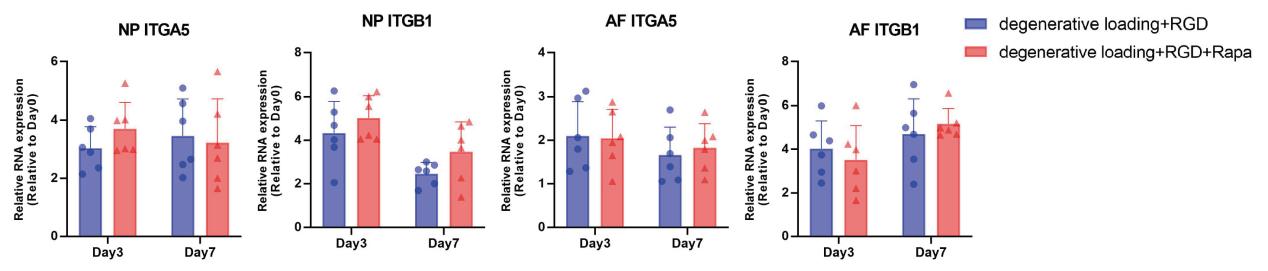


**Supplementary figure 2. Analyses of ITGA5 and ITGB1 RNA expression in IVDs under degenerative loading conditions (with RGD or RGD+Rapa).**

qPCR analysis of the relative mRNA expression of integrin subunit genes (ITGA5, ITGB1) in NP and AF tissues at Day 3 and Day 7. Groups compared: degenerative loading with RGD treatment (degenerative loading+RGD) and degenerative loading with combined RGD and rapamycin treatment (degenerative loading+Rapa). Data are expressed as mean ± SD (n = 6). Statistical significance: *p < 0.05.


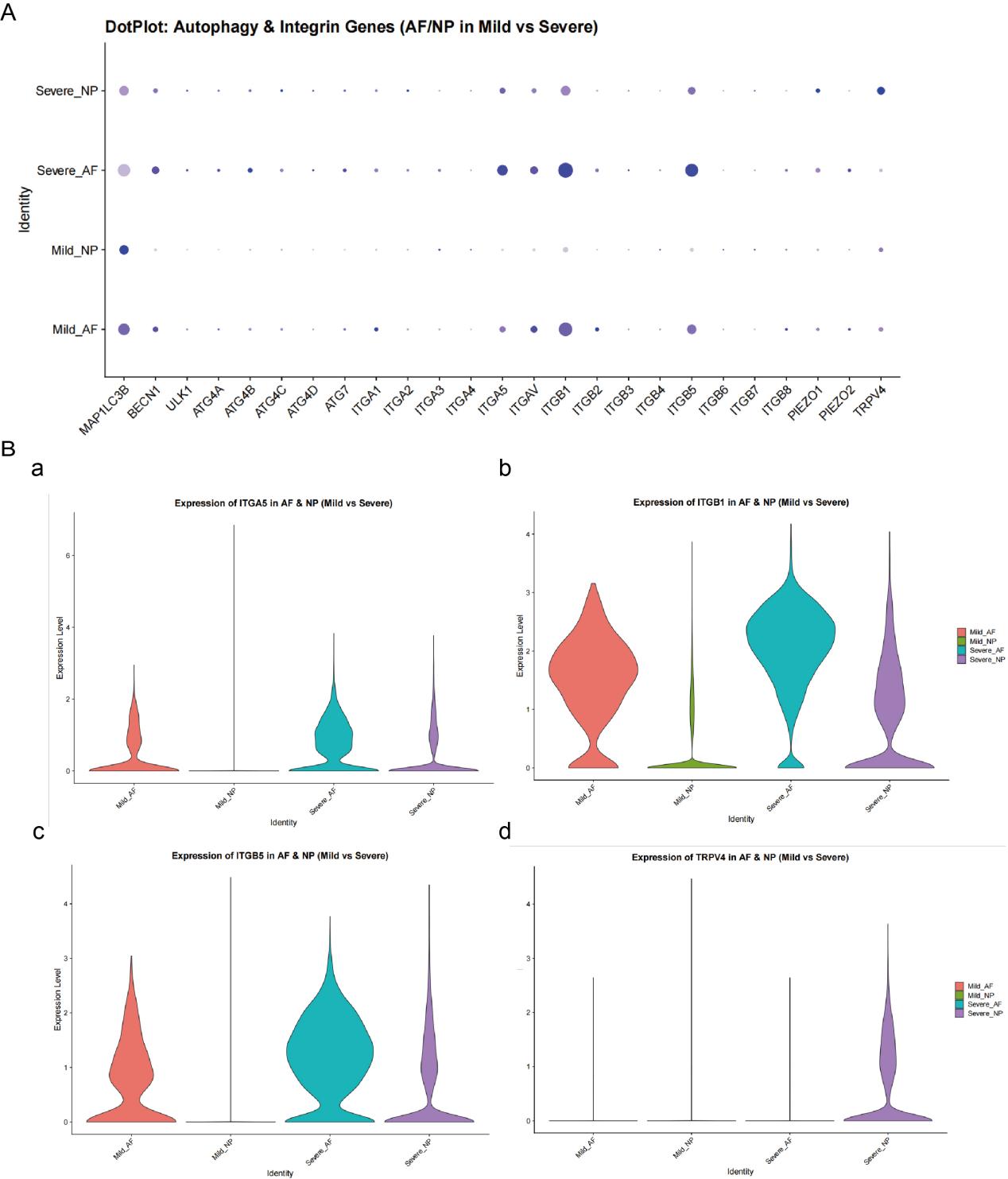


**Supplementary figure 3. Analyses of autophagy-related and integrin gene expression in IVDs based on GSE251686 public database sequencing data (mild vs severe degenerative conditions).**

(A) Dot plot showing the expression of selected autophagy-related and integrin genes across NP and AF tissues under mild versus severe degenerative conditions. Dot color indicates the average expression level, and dot size represents the percentage of expressing cells. (B) Violin plots detailing the expression distribution of four representative genes: (a) ITGA5, (b) ITGB1, (c) ITGB5, and (d) TRPV4, in NP and AF tissues under mild and severe conditions. All data were generated from scRNA-seq analysis of IVD tissues and visualized via bioinformatics tools. Data are presented as specified in individual subplots. Abbreviations: TRPV4, transient receptor potential cation channel subfamily V member 4.


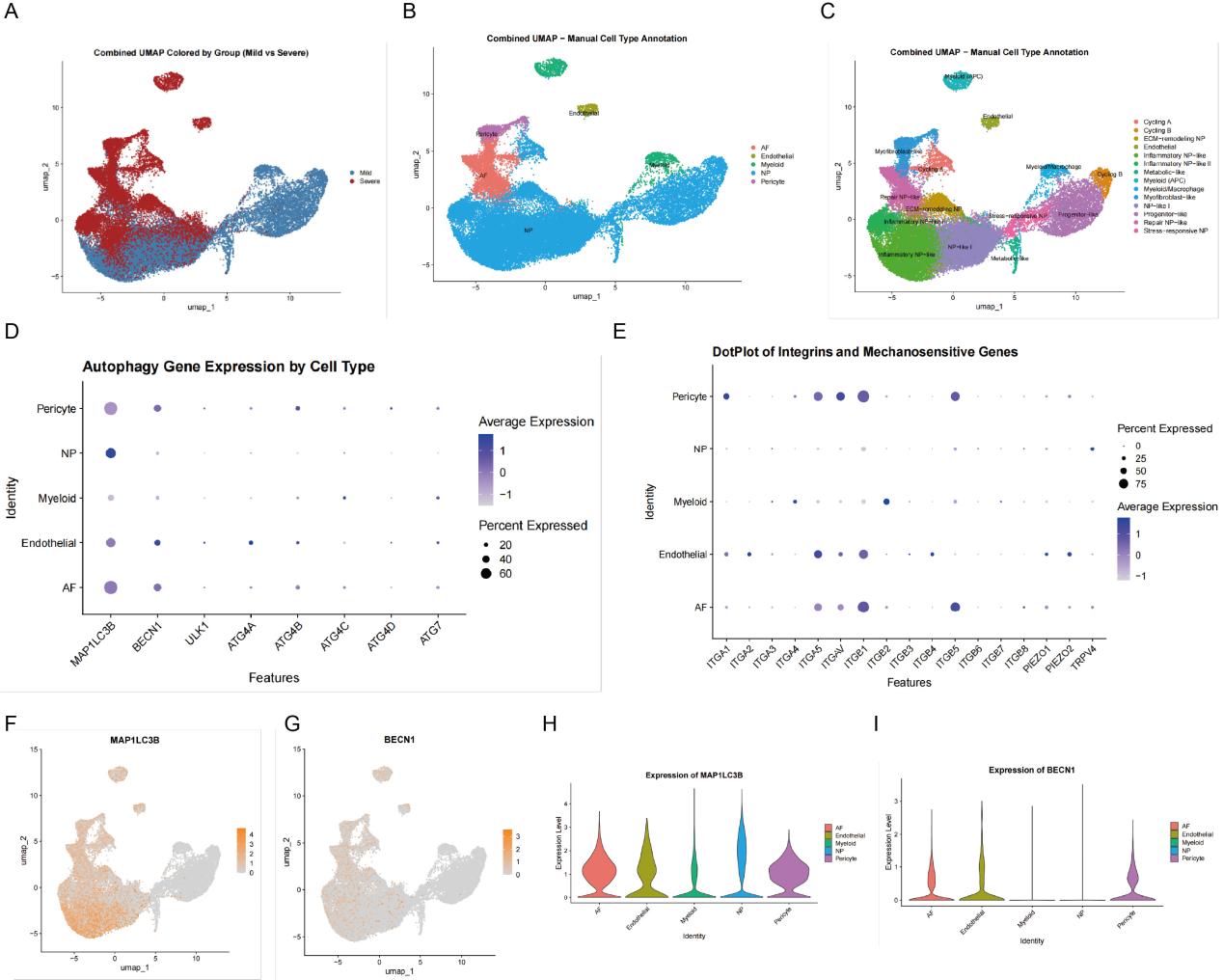


**Supplementary figure 4. Single-cell RNA sequencing analysis of cell types, autophagy-related genes, and integrin/mechanosensitive genes in intervertebral discs (IVDs).**

(A) UMAP visualization of all cells, colored by degenerative grade (Mild vs Severe). (B, C) UMAP visualizations annotated by cell type: (B) major cell classes; (C) detailed cell subtypes. (D, E) Dot plots visualizing the expression of (D) autophagy-related genes (e.g., MAP1LC3B, BECN1, ULK1) and (E) integrin/mechanosensitive genes across the major cell types. In both dot plots, color intensity represents the average expression level, and dot size corresponds to the percentage of expressing cells. (F, G) Feature plots (UMAP) showing the expression distribution of (F) MAP1LC3B and (G) BECN1. (H, I) Violin plots showing the expression levels of (H) MAP1LC3B and (I) BECN1 across different cell types.All data were generated from scRNA-seq analysis of IVD tissues and visualized via bioinformatics tools. Data are presented as specified in individual subplots. Abbreviations: UMAP, Uniform Manifold Approximation and Projection; MAP1LC3B, microtubule associated protein 1 light chain 3 beta; BECN1, beclin 1.
